# Supplementary material for: Late Embryogenesis Abundant Protein–Client Protein Interactions
Source: Plants (Basel). 2020 Jun 29;9(7):814. doi: 10.3390/plants9070814 (PMC7412488; doi:10.3390/plants9070814)
Supplement: Supplementary file 1 [file plants-09-00814-s001.zip › FINAL SUPPLEMENTARY/plants-833951 - supplementary tables for proofreading_ABDedits.docx]

**Table S1.** Attributes of *Arabidopsis thaliana* LEAPs, any client proteins identified to date, and post-translational modifications. Mutant or overexpression phenotypes for those of the 51 LEAPs in Arabidopsis for which a phenotype has been reported. Additionally, all instances of phenotypes for any Arabidopsis LEAP overexpressed non-con-specifically is presented. In those instances where a mutant phenotype occurs, tissue distributions and subcellular residences were acquired to assess if complementary LEAPs might be available and their occurrence, or lack thereof, is recorded in the Mutant phenotype column.

| **^∆^LEAP ID**  **Gene ID GID**  **[group]** | **Other Symbols, unusual attributes.**  **Structure available**  **Known interacting proteins** | ****A.t.* LEAP Subcellular compartment(s)**  **^¥^(PEST)**  **Post-translational modification** | **^#^*A.t.* tissue distribution** | ***A.t.* Mutant phenotype(s)**  **^£^Complementary LEAPs (family, tissue, timing, subcellular locale).** | ***A.t.* OE phenotype(s)** | **Arabidopsis LEAP OE phenotype(s) in Non-*A.t.* species** |
| --- | --- | --- | --- | --- | --- | --- |
| 1 At1g01470  [LEA_2] | Light stress-regulated 3, lsr3, AtLEA14 **[169].**  **Binds F-BOX ATPP2-B11**  **At1g80110.** [170] | cytosol  cytosol  **Phosphorylated. [163]**  **1 site.** | Mesophyll, root, guard cells, trichomes, stem epidermis, hypocotyl  Carpel, cauline leaf, collective leaf structure, cotyledon, flower, flower pedicel, guard cell, hypocotyl, inflorescence meristem, leaf apex, leaf lamina base, petal, petiole, plant embryo, pollen, root, seed, sepal, shoot apex, shoot system, stamen, stem, vascular leaf |  | Enhanced NaCl tolerance. [114] | Enhanced desiccation/ rehydration tolerance in yeast (*Saccharomyces cerevisiae*). [171] |
| 2 At1g02820  [LEA_3] | ATLEA3, LATE EMBRYOGENESIS ABUNDANT 3, LATE EMBRYOGENESIS ABUNDANT 38, LEA2, LEA3.  Histone modified by SDG8 (SET DOMAIN GROUP 8) EMF2 (EMBRYONIC FLOWER 2) to silence this gene after seed maturation. [146] | cytosol  cytosol  chloroplast, cytosol, mitochondrion, nucleus  **(PEST)** | Guard cells, pollen tubes, mesophyll, stem epidermis, leaves, trichomes  Carpel, cauline leaf, collective leaf structure, cotyledon, flower, guard cell, inflorescence meristem, leaf lamina base, petal, pollen, pollen tube cell, sepal, shoot system, stamen, stem, synergid, vascular leaf | Reduced drought tolerance and oil content. Reduced ROS scavenging. [172]  **No LEA3s.** | Enhanced drought tolerance, photosynthetic efficiency, and reduced ROS. [172] |  |
| 3 At1g03120  [SMP] |  | cytosol  cytoplasm, cytosol, nucleolus, nucleus | Procambium |  |  |  |
| 4 ^ç^At1g20440  [dehydrin] | ATCOR47, COLD-REGULATED 47, COR47, RD17.  **Actin binding [108].**  **Forms homodimers, binds dehydrins At1g20450, At5g66400,**  **Binds MIP Aquaporin AtPIP2B At2g37170.** [73,89] | cytosol  cytosol, membrane, nucleus  **(PEST)**  **Phosphorylated. [163]**  **7 sites.**  **Phosphorylation provides Ca binding capacity. [173]**  **Sumoylated. [174]** | Root, mesophyll, guard cells, trichomes, stem, rib meristem, leaves, hypocotyl, stigma, ovary, central zone, peripheral zone, xylem  Carpel, cauline leaf, collective leaf structure, cotyledon, flower, flower pedicel, guard cell, hypocotyl, inflorescence meristem, leaf apex, leaf lamina base, petal, petiole, plant embryo, pollen, root, rosette leaf, seed, sepal, shoot apex, shoot system, stamen, stem, trichome, vascular leaf. |  | With At5g66400, improved cold tolerance. [93] | Inhibits growth of *E. coli*. [175]  Enhanced desiccation/ rehydration tolerance in yeast (*S. cerevisiae*). [171] |
| 5 At1g20450  [dehydrin] | EARLY RESPONSIVE TO DEHYDRATION 10, ERD10, LOW TEMPERATURE INDUCED 29, LOW TEMPERATURE INDUCED 45, LTI29, LTI45  **[176]**  **Actin binding. [108]**  **Forms homodimers, binds dehydrins At1g20440 At5g66400; binds MIP Aquaporin AtPIP2B.** [73,89] | cytosol  cytoplasm, cytosol, membrane, nucleus, plasmodesma  **(PEST)**  **Phosphorylated by SNRK2.10. [83].**  **Phosphorylated. [163]**  **10-15 sites.**  **Phosphorylation provides Ca binding capacity. [173]** | **^€^Unstressed: R,S: v.t.; R: root tip; L,F: no staining. Chilled: R,S,F: general staining; R,S,L,F: v.t.; L: mesophyll. [177].**  Mesophyll, root, guard cells, trichomes, leaves, stem epidermis, rib meristem, stigma, central zone, ovary, hypocotyl, peripheral zone, xylem.  Carpel, cauline leaf, collective leaf structure, cotyledon, flower, flower pedicel, guard cell, hypocotyl, inflorescence meristem, leaf apex, leaf lamina base, petal, petiole, plant embryo, plant sperm cell, pollen, root, rosette leaf, seed, sepal, shoot apex, shoot system, stamen, stem, vascular leaf | More damage to leaves in cold; slower germination. [178]  TAIR: Under low temperature growth conditions (12°C with continuous low light) comparison of SALK_087789C to Columbia wild type resulted in significantly more rosette growth in the mutant (P<0.0001; rosette diameter 3.07cm:2.38cm). [Stapleton and Allery, personal communication Bio340 Fall 2009 (10-DEC-09)](https://www.arabidopsis.org/servlets/TairObject?id=501735552&type=communication).  **Acidic dehydrins.** | With At3g50970, improved cold tolerance. [93] | Inhibits growth of *E. coli*. [175]  Enhanced desiccation/ rehydration tolerance in yeast (*S*. *cerevisiae*). [171] |
| 6 At1g32560  [LEA_1] | ATLEA4-1, LATE EMBRYOGENESIS ABUNDANT 4-1, LEA4-1 | cytosol  cytosol, plasmodesma. | Torpedo – meristem, globular – basal, torpedo – basal, cork Col-0.  Guard cell, stem | **Caution**: Two LEA4 genes are downregulated: a-miR 4-1/2 *lea6/lea18*- reduced seed germination on NaCl (some negative effect on just MS and mannitol); *lea6/lea18* poorer recovery after drought; *lea6/lea18* reduced seed set. [51]  **LEA1s (Guard cell, stem, LEA46).** |  |  |
| 7 At1g52690  [LEA_4] |  | cytosol  cytosol  **Phosphorylated. [163]**  **4 sites.**  mRNA decay, in part, relies on VARICOSE and TRIDENT decapping enzymes. [179] | Guard cells, mesophyll, root, quiescent center, trichomes, central zone  Carpel, collective leaf structure, flower, pollen, root, sepal, stamen, stem, vascular leaf |  | Superior leaf water retention. Superior completion of germination on NaCl or mannitol. ABA hypersensitive. [180] | Imparts resistance to cold stress in *E. coli*. [180]  Enhanced desiccation/ rehydration tolerance in yeast (*S*. *cerevisiae*). [171] |
| 8 At1g54410  [dehydrin] | ATHIRD11, DEHYDRIN 11KDA, HIRD11**.**  **Cytosol to plasma membrane upon interaction with AtPXL1 LRR-RLK.** [79] | cytosol  cytosol, nucleus  **Phosphorylated by PXL1 (AT1G08590). [79]**  **Phosphorylated. [163]**  **5-7 sites.**  **Phosphorylated in vivo. [181]**  **Sumoylated. [174]** | Guard cells, trichomes, stem epidermis, root, leaves, pollen, hypocotyl, ovary, mesophyll, peripheral zone, quiescent center, stigma, xylem, central zone, rib meristem  Carpel, cauline leaf, collective leaf structure, cotyledon, flower, flower pedicel, guard cell, hypocotyl, inflorescence meristem, leaf apex, leaf lamina base, petal, petiole, plant embryo, pollen, pollen tube cell, root, rosette leaf, seed, sepal, shoot apex, shoot system, stamen, stem, vascular leaf |  |  |  |
| 9 At1g72100  [LEA_4] |  | pexophagosome  chloroplast, integral component of membrane, pexophagosome  **Phosphorylated. [163]**  **4 sites.** | Plant sperm cell |  |  |  |
| 10 At1g76180  [dehydrin] | EARLY RESPONSE TO DEHYDRATION 14, ERD14  **Phi9 GLUTATHIONE-S-TRANSFERASE9 (GSTF9; At2g30860).** [76] | cytosol  chloroplast, cytosol, membrane, mitochondrion, nucleus, plasma membrane  **(PEST)**  **Phosphorylated by SNRK2.10 (**[**AT1G60940**](https://www.arabidopsis.org/servlets/TairObject?name=AT1G60940&type=locus)**). [83]**  **Phosphorylated. [34]**  **5 sites.**  **Phosphorylation provides Ca binding capacity. [173]**  **Phosphorylated. [163]**  **5 sites.**  **Acetylated. [182]** | **^€^Unstressed: R, S, L, F: v.t.; R: root tip. Chilled: RS: general staining; L: mesophyll + v.t.; F: v.t. [177]**  Carpel, cauline leaf, collective leaf structure, cotyledon, flower, flower pedicel, guard cell, hypocotyl, inflorescence meristem, leaf apex, leaf lamina base, petal, petiole, plant embryo, pollen, pollen tube cell, portion of vascular tissue, root, root tip, rosette leaf, seed, sepal, shoot apex, shoot system, stamen, stem, vascular leaf | H_2_O_2_ hyper-accumulation. [76]  **Acidic dehydrins.** | H_2_O_2_ hypo-accumulation. [76] |  |
| 11 At2g03740  [LEA_4] |  | plastid  chloroplast | Torpedo stage embryo; ABA treated mesophyll cells  Collective leaf structure, flower, petal, sepal |  |  |  |
| 12 At2g03850  [LEA_4] |  | plastid  chloroplast | Torpedo stage embryo; ABA treated mesophyll cells; quiescent center; ovary tissue  Carpel, collective leaf structure, endosperm, flower, petal, pollen, sepal, stamen, stem  **Lateral root formation. [183]** |  |  |  |
| 13 At2g18340  [LEA_4] |  | endoplasmic reticulum  endoplasmic reticulum, extracellular region, vacuole | ABA treated mesophyll cells; quiescent center; ovary tissue; globular, heart, torpedo stage embryo; root endodermis and stele  Collective leaf structure, flower, petal, plant sperm cell, pollen |  |  |  |
| 14 At2g21490  [dehydrin] | LEAM | cytosol  cytosol, nucleus  **(PEST)**  **Phosphorylated. [163]**  **1 site.** | Heart and torpedo stage embryo; trichomes; root; peripheral zone.  Collective leaf structure, flower, guard cell, petal, sepal | RNAi reduced seed longevity; reduced completion of seed germination on NaCl **Caution**: RNAi also downregulated 2 other DHNs; LEA34: XERO1 At3g50980 and LEA51: RAB18 At5g66400). [11].  **Acidic dehydrins.** |  | Enhanced desiccation/rehydration tolerance in yeast (*S*. *cerevisiae*). [171] |
| 15 At2g23110  [PvLEA_18] |  | cytosol  cytoplasm, cytosol | Globular, heart, and torpedo stage embryo; root; trichomes; guard cells; quiescent center; mature and tricellular pollen grain; peripheral zone; stigma and ovary tissue; stem epidermis; central zone.  **Hypocotyl. [184]** |  |  |  |
| 16 At2g23120  [PvLEA_18] |  | cytosol  cytosol, plasma membrane, vacuole  Apoplast [185]  **(PEST)**  **Phosphorylated. [163]**  **4 sites.** | Guard cells; central zone; rib meristem; mesophyll cells; peripheral zone; trichomes; root epidermis, endodermis and stele; quiescent center; leaves; stem; stigma and ovary tissue; mature and tricellular pollen, pollen tube; hypocotyl; cork  Carpel, cauline leaf, collective leaf structure, cotyledon, flower, flower pedicel, guard cell, hypocotyl, inflorescence meristem, leaf apex, leaf lamina base, petal, petiole, plant embryo, pollen, pollen tube cell, root, rosette leaf, seed, sepal, shoot apex, shoot system, stamen, stem, vascular leaf |  |  |  |
| 17 At2g33690  [PvLEA_18] |  | cytosol  cytosol | Bi- and Tri-cellular pollen.  Collective leaf structure, flower, petal, pollen |  |  |  |
| 18 At2g35300  [LEA_1] |  | cytosol  cytosol | ABA treated guard cells; trichomes; torpedo stage embryo  Flower, stamen  **Mature pollen, pollen tube. [186]**  **Hypocotyl [184]** | **Caution**: Two LEA4 genes are downregulated: a-miR 4-1/2 *lea6/lea18*- reduced seed germination on NaCl (some negative effect on just MS and Mannitol); *lea6/lea18* poorer recovery after drought; *lea6/lea18* reduced seed set. [51]  Pollen tube rupture. [186]  **LEA1s (Stem, stamen, flower, pollen, LEA46).** |  |  |
| 19 At2g36640  [LEA_4] | AtECP63 | cytosol  cytosol | ABA treated guard cells; globular, heart and torpedo stage embryo; root; quiescent center; trichomes  Fruit, seed |  |  |  |
| 20 At2g40170  [LEA_5] | ATEM6  ARABIDOPSIS EARLY METHIONINE-LABELLED 6, ATEM6, EARLY METHIONINE-LABELLED 6, EM6, GEA6, LATE EMBRYOGENESIS ABUNDANT 6.  mRNA from which is bound by STRESS ASSOCIATED RNA-BINDING PROTEIN1. [153] | cytosol  cytoplasm, cytosol  **Phosphorylated. [163]**  **1 site.** | ABA treated guard cells; heart stage embryo.  Guard cell, plant embryo, seed. hypocotyl | Seed dehydration increased at distal end of silique. [187,188]  **LEA5s (Embryo, seed, LEA35).** | AtMYB118 OE lines up-regulates many *LEAPs*, including At2g40170 (second greatest) and has the same seed dehydration phenotype as At2g40170 KO. [189] |  |
| 21 At2g41260  [AtM] | M17 | vacuole  extracellular region, vacuole | Ovary tissue; trichomes; ABA treated mesophyll cells.  Cotyledon |  |  |  |
| 22 At2g41280  [AtM] | M10 | vacuole  endoplasmic reticulum, extracellular region, vacuole | Mesophyll cells; globular, heart, and torpedo stage embryos; trichomes |  |  |  |
| 23 At2g42530  [LEA_4] | COR15b | plastid  chloroplast, chloroplast envelope, chloroplast stroma, cytosol  **Phosphorylated. [163]**  **1 site.** | Carpel, cauline leaf, collective leaf structure, cotyledon, flower, flower pedicel, guard cell, hypocotyl, inflorescence meristem, leaf apex, leaf lamina base, petal, petiole, plant embryo, pollen, rosette leaf, seed, sepal, shoot apex, shoot system, stamen, stem, vascular leaf | None discerned, compensated by COR15A up-regulation. [190]  RNAi of both At2g42530 and At2g42540 results in smaller rosette, delayed flowering. [115].  **LEA4s (LEA9, 11, 12).** | Decreased NaCl tolerance. [190]  Enhanced chloroplast freezing tolerance  Smaller rosette, later flowering. [115] |  |
| 24 At2g42540  [LEA_4] | COR15a  Light up-regulated. [191]  **Forms homo-oligomers. Binds both the large- and small-subunits of RUBISCO. [103]** | plastid  chloroplast, chloroplast envelope, chloroplast stroma, cytosol  **Phosphorylated. [163]**  **2 sites.** | Carpel, cauline leaf, collective leaf structure, cotyledon, flower, flower pedicel, guard cell, inflorescence meristem, leaf apex, leaf lamina base, petal, petiole, plant embryo, pollen, portion of vascular tissue, rosette leaf, seed, sepal, shoot apex, shoot system, stamen, stem, vascular leaf | RNAi of both At2g42530 and At2g42540 results in smaller rosettes, delayed flowering. [115].  **LEA4s (LEA9, 11, 12).** | Enhanced chloroplast and protoplast freezing tolerance. [106]  Improved protoplast freezing tolerance in the range -5 to -7°C. [192]  Decreased NaCl, mannitol tolerance. [191]  Smaller rosette, later flowering. [115] |  |
| 25 At2g42560  [LEA_4] |  | cytosol  cytosol, nucleus  **(PEST)**  **Phosphorylated. [163]**  **2 sites.** | Seed |  |  |  |
| 26 At2g44060 [LEA_2] | Red light protein accumulation through CRYPTOCHROMES. [193]  Binds GIGANTEA. [194] | cytosol  Golgi apparatus, cytosol, nucleus, plasma membrane, plasmodesma  **Phosphorylated. [195].**  **Phosphorylated. [163]**  **1 site.** | Carpel, cauline leaf, collective leaf structure, cotyledon, cultured plant cell, flower, flower pedicel, guard cell, hypocotyl, inflorescence meristem, leaf apex, leaf lamina base, petal, petiole, plant embryo, plant sperm cell, pollen, root, rosette leaf, seed, sepal, shoot apex, shoot system, stamen, stem, vascular leaf |  |  |  |
| 27 At2g46140 [LEA_2] | **Released: 2005-04-05**  **Deposition Author(s): Center for Eukaryotic Structural Genomics (CESG), Song, J., Tyler, R.C., Lee, M.S., Markley, J.L. DOI: 10.2210/pdb1YYC/pdb**  **BMRB: 6515.**  **[196]** | cytosol  cytosol, plasma membrane | Carpel, cauline leaf, collective leaf structure, cultured plant cell, flower, flower pedicel, guard cell, hypocotyl, inflorescence meristem, leaf apex, leaf lamina base, petal, petiole, plant embryo, plant sperm cell, pollen, pollen tube cell, root, seed, sepal, shoot apex, shoot system, stamen, stem, vascular leaf |  |  | Enhanced desiccation/rehydration tolerance in yeast (*S*. *cerevisiae*). [171] |
| 28 At3g02480  [LEA_4] | ABA-RESPONSE PROTEIN, ABR.  Epigenetically silenced through HDA19- SCL15 joint action in the embryo to seedling phase transition. [144] | cytosol  cytosol  **Phosphorylated. [163]**  **2 sites.** | Carpel, collective leaf structure, flower, petal, pollen, pollen tube cell, portion of vascular tissue, root, sepal, stamen, vascular leaf | Premature leaf senescence phenotype. [197].  **LEA4s (LEA7, 39, 40).** | Marked delay in dark-induced leaf senescence phenotypes. [197] |  |
| 29 At3g15670  [LEA_4] |  | cytosol  cytosol, nucleus  **Phosphorylated. [163]**  **2 sites.** | Mature, dehydrated seed |  |  | Imparts resistance to cold stress in *E. coli*. [180]  Enhanced desiccation/rehydration tolerance in yeast (*S*. *cerevisiae*). [171] |
| 30 At3g17520  [LEA_4] | Epigenetically silenced by CLF/SWN. [145]  Also histone modified by SDG8 (SET DOMAIN GROUP 8) EMF2 (EMBRYONIC FLOWER 2). [146] | Endoplasmic reticulum  Endoplasmic reticulum, extracellular region, vacuole  **Phosphorylated. [163]**  **1 site.** | Cultured plant cell, seed, vascular leaf  **Hypocotyl [184]** |  |  |  |
| 31 At3g22490  [SMP] | RAB28 | cytosol  cytoplasm, cytosol, nucleolus, nucleus  **nucleus and nucleolus [198]** | **^€^Mature embryo provascular tissues, seed coat outer integument and embryo and silique epidermis [199]**  Procambium | Decreased seed dormancy. [102]  **SMPs (procambium LEA3; all LEA32, 49, 50).** | Enhanced germination rate on H_2_O, LiCl, NaCl, and sorbitol. Greater seedling survival on LiCl. [198] |  |
| 32 At3g22500  [SMP] | AtECP31 | cytosol  cytoplasm, cytosol, nucleus | Seed |  |  |  |
| 33 At3g50970  [dehydrin] | XERO2; LTI30 | cytosol  cytosol, membrane  **Phosphorylated. [61]** | **^€^Unstressed: R, S, L, F: no staining. Chilled: R, S, L, F: v.t. and R: general; F: pollen sacs. [177]**  Cauline leaf, collective leaf structure, flower, guard cell, hypocotyl, inflorescence meristem, leaf apex, petal, petiole, plant embryo, portion of vascular tissue, root, rosette leaf, sepal, shoot apex, stamen, stem, vascular leaf | ABA hyposensitive. Decreased drought tolerance. [200]  **Neutral/basic dehydrins (LEA8, 34, 51).** | With At1g20450, improved cold tolerance. [93]  ABA hypersensitive. Increased drought tolerance. [200] |  |
| 34 At3g50980  [dehydrin] | XERO1 | cytosol  cytoplasm, cytosol | Collective leaf structure, inflorescence meristem, petal, seed, shoot apex | RNAi reduced seed longevity; reduced completion of seed germination on NaCl. **Caution**: RNAi also downregulated 2 other DHNs; LEAM At2g21490 and RAB18 At5g66400. [11]  **Neutral/basic dehydrins (LEA8, 33).** |  |  |
| 35 At3g51810  [LEA_5] | AtEM1.  mRNA from which is bound by STRESS ASSOCIATED RNA-BINDING PROTEIN1. [153] | cytosol  cytoplasm, cytosol | Plant embryo, seed |  |  |  |
| 36 At3g53040  [LEA_4] | Repressed by HIGH-LEVEL EXPRESSION OF SUGAR INDUCIBLE  GENE2; HSI2)/VAL1 (VP1/ABI3-LIKE1) partially by H3K27me3. [201] | cytosol  cytosol  **(PEST)**  **Phosphorylated. [163]**  **1 site.** | Flower, flower bud, seed | Greater capacity to re-introduce desiccation tolerance in seeds that had completed germination. Potentially due to over-compensation by a close paralog. [202]  **LEA4s (flower LEA7, 28, 39, 40; seed LEA19, 25, 29)** |  |  |
| 37 At3g53770  [LEA_3] |  | mitochondrion  cytosol | Flower |  |  |  |
| 38 At4g02380  [LEA_3] | AtLEA5; AtLEA3; Senescence associated gene 21 (SAG21). | mitochondrion mitochondrion Chloroplast, mitochondrion  **Acetylated. [182]** | Carpel, cauline leaf, collective leaf structure, cotyledon, flower, flower pedicel, guard cell, hypocotyl, inflorescence meristem, leaf apex, leaf lamina base, petal, petiole, plant embryo, pollen, pollen tube cell, root, sepal, shoot apex, shoot system, stamen, stem, vascular leaf | Shorter root hair length. Reduced rosette FW and leaf number @10 wk; fewer d to bolting and flowering; smaller mean lateral root+ lateral root primordia as a function of primary root length. [203]  **LEA3s (Mito/flower LEA37; all LEA41).** | Improved drought tolerance. Increased oxidative stress tolerance. [203].  Longer root hair length. Increased leaf number @10 wk; more d to bolting and flowering; larger mean lateral root+ lateral root primordia as a function of primary root length. [204]. | Compliments the H_2_O_2_ sensitivity of yeast (*S*. *cerevisiae*) delta *yap1* mutant. [204] |
| 39 At4g13230  [LEA_4] |  | cytosol  cytosol | Carpel, collective leaf structure, flower, petal, pollen, stamen |  |  |  |
| 40 At4g13560  [LEA_4] | Unfertilized embryo sac 15. | cytosol  cytosol  Apoplastic from germinating pollen tubes. [205].  Pollen proteome [206].  **Phosphorylated. [163]**  **4 sites.** | Carpel, cauline leaf, collective leaf structure, cotyledon, flower, guard cell, leaf apex, leaf lamina base, petal, plant embryo, pollen, pollen tube cell, sepal, stamen, stem, vascular leaf | Defects in pollen tube attraction. [207]  **LEA4s (flower LEA7, 19, 28, 36; all LEA39).** |  | Enhanced desiccation/rehydration tolerance in yeast (*S*. *cerevisiae*). [171] |
| 41 At4g15910  [LEA_3] | AtD121  The mRNA of which is bound by RNA BINDING PROTEIN1 (RBP1). [154] | Mitochondrion  Chloroplast, mitochondrion | Carpel, cauline leaf, collective leaf structure, cotyledon, flower, flower pedicel, guard cell, hypocotyl, inflorescence meristem, leaf apex, leaf lamina base, petal, petiole, plant embryo, pollen, pollen tube cell, root, seed, sepal, shoot apex, shoot system, stamen, stem, vascular leaf |  |  |  |
| 42 At4g21020  [LEA_4] |  | mitochondrion and plastid  mitochondrion, plasma membrane, plastid  **Phosphorylated. [163].**  **2 sites.** | Seed development |  |  |  |
| 43 At4g36600  [LEA_4] |  | Endoplasmic reticulum  Endoplasmic reticulum, nucleus  **Phosphorylated. [163]**  **4 sites.** | Collective leaf structure, flower, petal, plant sperm cell, pollen, pollen tube cell |  |  |  |
| 44 At4g38410  [dehydrin] |  | cytosol  cytosol, membrane | Hypocotyl, root, stem |  |  |  |
| 45 At4g39130  [dehydrin] |  | cytosol  cytosol | Carpel, collective leaf structure, flower, petal, pollen, stamen |  |  |  |
| 46 At5g06760  [LEA_1] | AtLEA4-5  Transcription of which is repressed by MYB44 in the absence of stress. [147]  mRNA decay, in part, relies on VARICOSE and TRIDENT decapping enzymes. [179] | cytosol  cytosol  **Phosphorylated. [163]**  **1 site.** | Carpel, collective leaf structure, flower, guard cell, petal, pollen, stamen, stem, vascular leaf | Reduced germination on NaCl and mannitol; reduced seed set. [51]  **LEA1s (Stem hypocotyl LEA6; Guard cell, pollen, stamen, flower, hypocotyl LEA18).** | Enhanced drought tolerance and re-watering recovery. [51] |  |
| 47 At5g27980  [SMP] |  | cytosol  cytoplasm, cytosol, nucleus**.**  **Phosphorylated. [163]**  **1 site.** | Collective leaf structure, flower, petal, pollen, pollen tube cell, sepal |  |  |  |
| 48 At5g44310  [LEA_4] | Closest ortholog to *Pisum sativum* LEAM for which structure, when desiccated, is known.  **Tolleter et al.** **[27]** | mitochondrion and plastid | Cotyledons, seeds | NaCl hypersensitive. [202]  **LEA4s (plastid LEA11, 12, 23, 24; all LEA42)** |  |  |
| 49 At5g53260  [SMP] |  | cytosol  cytosol, nucleus | Mature, dehydrated seeds |  |  |  |
| 50 At5g53270  [SMP] |  | cytosol  cytoplasm, cytosol, nucleus  **Phosphorylated. [163].**  **1 site.** | Mature, dehydrated seeds |  |  |  |
| 51 At5g66400  [dehydrin] | RAB18  **Forms homodimers, binds dehydrins At1g20440, At1g20450,**  **Binds MIP Aquaporin AtPIP2B.** [73,89] | cytosol  cytosol  **Phosphorylated. [163]**  **1 site.** | **^€^Unstressed: R, no staining. S, L, F: guard cells. S: v.t. ABA: R,S,L,F: v.t. and R,S,F: general; L: mesophyll, guard cell. [177]**  Cauline leaf, collective leaf structure, cotyledon, flower, guard cell, inflorescence meristem, leaf apex, leaf lamina base, petal, petiole, pollen, seed, sepal, shoot system, stamen, stem, vascular leaf | RNAi reduced seed longevity; reduced completion of seed germination on NaCl. **Caution**: RNAi also downregulated 2 other DHNs; LEAM At2g21490 and XERO1 At3g50980. [11]  **Neutral/basic dehydrins (LEA8, 33, 34)** | With At1g20440 Improved cold tolerance. [93] |  |

^∆^The LEAP numbering, family identity scheme, and coloration is according to [64].

*For Arabidopsis sub-cellular compartmentalization, see Candat et al. [208]; Avelange-Macherel et al. [209]; or **TAIR [210]**.

**^¥^**PEST domain prediction used epestfind (http://emboss.bioinformatics.nl/cgi-bin/emboss/epestfind).

^#^For Arabidopsis predominant tissue distribution, see **^€^protein localization using immune-staining ([177]; R: Root; S: Shoot; L: Leaf; F: Flower. general = western signal throughout tissue; v.t. = western signal in vascular tissue)**; eFP at the University of Toronto BAR site (**mRNA only**) [211]; **TAIR (mRNA only)**; Bardou et al. [183]; and Lorrai et al. [184].

^£^Opportunity for complementation of mutant phenotypes assumed based on: 1) LEAPs from the same family; 2) identical sub-cellular localization; 3) tissue distribution of mRNA (at least) from the preceding two columns; and 4) similar developmental/induction timing (From the BAR site Expression angler). No overlapping LEAP distribution. Overlapping LEAP distribution.

**^ç^**Acidic dehydrins; neutral or basic dehydrins.

Analyses used to generate this table are presented, in part, in Spreadsheet S1.

**Table S2.** Non-*Arabidopsis thaliana* LEAPs displaying phenotypes in any species.

| **Gene ID (GID)**  **[LEAP family]**  **<Protein identifier>** | **Species encoding the LEAP^1^** | **Mutant/RNAi/**  **Antisense/VIGS phenotype(s)** | **OE phenotype(s)** | **Heterologous OE phenotype(s)** | **References** |
| --- | --- | --- | --- | --- | --- |
| ApY_2_SK_2_  ApSK_3_  [dehydrin types as listed]  <Shotgun sequence drafts, ApY_2_SK_2_ (CL14770.Contig1) and ApSK3 (Unigene1977); CDS and protein in Figure 3 in [212]> | *Agapanthus praecox*  ssp. o*rientalis*  blue lily |  |  | *Arabidopsis thaliana* (Col-0) transformed with constitutively expressing GFP:dehydrin-fusion had reduced damage to the plasma membrane and lower ROS levels but higher antioxidant activity and photosynthesis capability upon salt (0.15 M NaCl), osmotic (0.3 M mannitol), cold and drought (withheld water for 15 d and rewatered for 12 d) stresses compared with the WT plants. | [212] |
| AcLEA  [group 3 LEA_4; pfam PF02987]  <GenBank cDNA KX852451> | *Amaranthus cruentus*  purple amaranth |  |  | *E*. *coli* which included the AcLEA in the vector display enhanced tolerance to oxidative stress (0.1, 0.5 and 1 mM H_2_O_2_), dehydration (tested with drying and resuspending prior to plating; as well as with sorbitol [0.4, 0.6 and 0.8 M]) and osmotic potential stress (5, 10 and 20% w/v PEG4000) as compared to the empty vector cultures. No more tolerant to NaCl (0.4, 0.6 and 0.8 M) than the empty vector *E*. *coli*. | [213] |
| AmCIP  [dehydrin-like]  <Undisclosed> | *Ammopiptanthus mongolicus*  evergreen broadleaf shrub |  |  | Ectopic expression in *E*. *coli* (ER2566) as either a N or C-terminal fusion to the vector’s chitin-binding and self-cleaving intein sequence, provided greater freezing tolerance relative to empty vector cultures under the same conditions, as determined by survival percentage (colony forming units) after freezing (liquid nitrogen 30 s) and thawing at a defined OD. *Nicotiana* sp. (tobacco) expressing *AmCIP* under control of the Arabidopsis *rd RD29* promoter increased freezing tolerance (higher seed germination percentage at 4 °C for 5 d; lower electrical conductivity of 8 wk-old plants after 24 h at -2 °C) relative to WT tobacco under the same conditions. | [214] |
| AmDHN132  AmDHN154  AmDHN 200  [dehydrin; KS-type]  <AmDHN132 GenBank CDS MH512938; AmDHN154 GenBank CDS MH512939; AmDHN 200 GenBank CDS MH512941> | *Ammopiptanthus mongolicus*  evergreen broadleaf shrub |  |  | Transgenic Arabidopsis for each dehydrin, as GFP fusion, were compared with empty vector plants in media with different additives. AmDHN132 consistently performed the best of the 3 dehydrins in NaCl (0.1, 0.15 and 0.2 M), osmoticum (0.1, 0.2 and 0.3 M mannitol), and after -5 °C for 4 h (which was the only stress that DHN154 did not also assist in resistance). | [215] |
| AmLEA  [LEA_3; pfam PF03242]  <GenBank cDNA AY843523> | *Ammopiptanthus mongolicus*  evergreen broadleaf shrub |  |  | As a chitin-binding fusion protein in *E*. *coli* (BL21), AmLEA increased survival of the bacteria when exposed to either 0 (on solid media for 2, 4, 6, 8, 10 or 12 d) or 50 °C (in liquid media for 0.5, 1, 1.5, 2 and 4 h and then plated on solid media) as compared to empty vector control cultures. | [216] |
| AdLEA  [atypical group 5C; LEA_2; pfam PF03168]  <GenBank partial cDNA GU223575> | *Arachis diogoi* Hoehne  (Syn. *A. chacoense*)  wild relative of cultivated peanut |  |  | Greater proline accumulation and higher *Fv*/*Fm* values (quantum yield of photosynthesis measurement) during drought stress (withholding water for 18 d) in the transgenic tobacco (*Nicotiana tabacum* cv. Samsun) plants as compared to untransformed plants under the same conditions. Longer roots and greater chlorophyll retention was observed as enhanced drought (10 and 12% PEG6000), osmotic (0.3 M sorbitol), NaCl (0.2 and 0.3 M), and oxidation (only chlorophyll retention; 10 μM MV) tolerance for both seedlings and as a disc from a mature leaf of the AdLEA-plants compared with WT under the same conditions. Increased gene expression of antioxidant enzymes in the transgenic plants compared with the WT plants under both normal and drought conditions. | [217] |
| AdDHN1  [dehydrin SK_2_-type; pfam PF00257]  <Gene ID Aradu.IF4XP.1 at peanutbase.com> | *Arachis duranensis*  wild peanut |  |  | Overexpression (eGFP vector) in Arabidopsis (Columbia) improved tolerance to both freezing (-18 °C for 1 h) and drought (8 d withholding water) but decreased tolerance to the biotic stress of *Meloidogyne incognita* nematode (root-knot) infection (measured by numbers of females in the root system 60 d post infection of 1000 juveniles). | [218] |
| AoDHN1  [dehydrin; YSK_2_ type; pfam PF00257]  <GenBank CDS KM652423> | *Avicennia officinalis*  mangrove tree |  |  | In *E*. *coli* slight growth advantage compared with empty vector control with no stress. Potentially additional superior growth under stressors of NaCl (0.4 M), mannitol (0.5 M), or PEG4000 (10%). | [219] |
| BdASR1  [ASR]  <Gene ID Bradi2g61590> | *Brachypodium distachyon*  strain Bd21  purple false brome |  |  | A pGBKT7 vector (yeast strain AH109) with the BdASR1 sequence could act as a transcriptional activator. BdASR-overexpressing tobacco (cv. Samsun), 5 wk-old plants, as compared to untransformed or vector control plants under the same conditions, were more tolerant to drought (30 d withholding water and 3 d rewatering; measured as a greater survival percentage), showing less H_2_O_2_, MDA and ion leakage but greater antioxidant (glutathione) and chlorophyll contents and enzyme activities (SOD and POD) that scavenge ROS. | [220] |
| BdASR4  [ASR; pfam PF02496]  <Gene ID Bradi4g24650> | *Brachypodium distachyon*  genotype Bd21, accession no. W636678  purple false brome |  | Constitutively expressed *BdASR4* *Brachypodium distachyon* plants were more drought tolerant (greater survival percentages after rewatering following 10 d drought), with higher antioxidant enzyme activities (SOD, CAT, POD, and APX) and greater RWC and chlorophyll content; but lower MDA content and less ion leakage than WT plants under the same conditions. The transgenic seeds were hypersensitivity to ABA (1 μM) in culture compared with WT seeds. |  | [221] |
| BjDHN2  BjDHN3  [dehydrin; SK_2_-type; pfam PF00257]  <BjDHN2 GenBank partial CDS DQ441470; BjDHN3 GenBank cDNA DQ441471> | *Brassica juncea*  Indian mustard | Antisense construct *bjdhn3* in *B. juncea* results in increased electrolyte leakage and MDA content upon exposure (in Hoagland solution) to CdCl_2_ (0.1 mM) and ZnCl_2_ (0.2 mM), relative to the empty vector control. [222] |  | Overexpression of either *BjDHN2* or *BjDHN3* in tobacco (cv. NC89) results in faster reduction of electrolyte leakage and MDA content after exposure (in Hoagland solution) to CdCl_2_ (0.1 mM) and ZnCl_2_ (0.2 mM) one the stress is removed relative to the empty vector control. [222]  Transgenic yeast (*S*. *cerevisiae* W303  (ura3-1, can-1-100, leu 2-3 112 trp 1-1, his 3-11, 15)) with pYES2 vector containing either dehydrin under the GAL1 promoter had a shorter lag period and better growth upon normal conditions after 48 h at -20 °C following quick freezing as compared to the empty vector control yeast under the same conditions. Both dehydrins with induction by galactose in the yeast improved growth in the presence of 1 M NaCl as compared to the empty vector in the cultured yeast. Tobacco transgenic plants constitutively overexpressing either dehydrin were also more cold- (4 °C for 10 d as leaf discs; greater chlorophyll retention) and NaCl-tolerant (cultured plantlets in the presence of 0.15 M and then recovery had RWC, electrolyte leakage, and MDA content that trended toward prestress amounts more quickly) the than the empty vector control plants under the same conditions. [222,223] | [222,223] |
| BnCOR25  [dehydrin; pfam PF00257]  <GenBank CDS HM187577> | *Brassica napus*  rape |  |  | Greater survival (as a percentage of 100 not stained blue by methylene blue after 48 h a -20 °C relative to those stored at 4 °C) for the yeast (*Schizosaccharomyces pombe*) only under induced conditions as compared to the empty vector yeast cultures. Arabidopsis plants with the dehydrin overexpressed had enhanced tolerance to cold (longer root lengths after returning to normal temperatures for 5 d following 2 d at 4 °C; greater retention of chlorophyll after 5 d at 4 °C) compared to WT plants under the same conditions. | [224] |
| BnLEA3  [group 3; pfam PF03242]  <BnLEA3.1 GenBank CDS KP315905; BnLEA3.2 GenBank CDS KP315906; BnLEA3.3 GenBank CDS KP315907; BnLEA3.4 GenBank CDS KP315908> | *Brassica napus*  rape | *B*. *napus* plants with an RNAi vector produced seeds of a decreased size (1000 seed weight) under either normal or drought conditions of the plants relative to those of WT plants or the complemented mutant. | Larger seed size, superior drought tolerance, relative to WT. Increased seed oil content. |  | [172] |
| BnLEA4-1  [group 4; LEA_1; pfam PF03760]  <GenBank CDS AY572958> | *Brassica napus*cv. Electrape |  |  | Superior survival in NaCl (0.8 M) and extreme temperature tolerance (4° or 48° C) of *E. coli* expressing *BnLEA4-1*. Qualitatively superior NaCl (0.15 M) and drought stress (15 d withholding water and 1 wk rewatering) tolerance in Arabidopsis ectopically expressing *BnLEA4-1*. | [225] |
| ME-LEAN4  [group 3]  <GenBank cDNA AB083361> | *Brassica napus*rape |  |  | *Lactuca sativa* (lettuce) ectopically expressing *LEAN4* exhibited superior NaCl (30, 50, 70 and 100 mM; hydroponic plants) and drought tolerance (10 d withholding water; plants in soil) to WT, as measured by greater plant height and FW. [226]  In *Brassica campestris* ssp. *pekinensis* (Chinese cabbage), ectopic expression of *ME-LEAN4* confers enhanced NaCl tolerance (90, 120, 150, 180 and 210 mM) relative to WT, as measured by the percentage of seed which completed germination on solid media. Qualitatively, the transgenic plants also seem to withstand drought better than the non-transgenic control plants. [227]  In *Raphanus sativus* L. (radish), plant length and FW of the transgenic plants were greater than the non-transgenic plants in the presence of mannitol (0.05, 0.1, 0.2, 0.3 and 0.4 M) or NaCl (0.05, 0.1, and 0.15 M). [126] | [126,226,227] |
| ROB5  [LEA3]  <Undisclosed> | *Bromus* *inermis* Leyss  cv. Manchar  bromegrass |  |  | The *Solanum tuberosum* (potato), canola (undisclosed) and flax (undisclosed) plants each transformed with an inducible promoter (COR78) driving ROB5 were more freezing, drought and heat tolerant. The canola seed were faster to emerge in the field and the plants faster to begin flowering than those from the WT plants. [228]  The ROB5-overexpressing potato (cv. Desiree) plants flowered earlier and grew taller. A COR78 promoter to drive RO5 expression were higher tuber yielding than untransformed potato plants across 5 experiments when drought was imposed at the beginning of tuber initiation. The same plants had greater freezing and heat tolerance as compared with WT plants. [130] | [130,228] |
| CsLEA7  [group 7]  <GenBank gene KC888958> | *Camellia sinensis* (L.) O. Kuntze  tea |  |  | *E. coli* (M15 pREP-4) ectopically expressing with the use of a pQE-30 UA vector, *CsLEA7* had greater growth even in LB media and with the additives of PEG8000 (10%), NaCl (0.8 M), H_2_O_2_ (0.027%) or 4°C than the vector control cultures. | [229] |
| CaDIL1  [group 3; LEA_4; pfam PF02987]  <Protein in Figure 1 in [230]> | *Capsicum annuum*  pepper | Using VIGs, 3 wk-old pepper plants had reduced drought tolerance (survival rate following 10 d withholding water and 2 d rewatering) and ABA sensitivity (larger stomatal aperature with application of 10 and 20 μM to leaf peels), relative to empty vector control plants. |  | In Arabidopsis ectopically expressing *CaDIL1*, the seeds completed germination to a lower percentage on ABA (0.75 μM) compared to seeds of WT plants. Those that did complete germination on either 0.75 or 20 μM ABA had shorter roots compared to those of WT. Enhanced drought tolerance (higher survival percentage following 14 d withholding water and 2 d rewatering) was observed for the transgenic plants relative to WT. | [230] |
| CaLEA  [Undisclosed]  <Undisclosed> | *Capsicum annuum*  “hot” pepper |  |  | In Chinese cabbage (cv. Seoul) ectopic expression of *CaLEA* resulted in superior tolerance to NaCl (0.25 M twice a wk; leaf chlorophyll content) or 45°C (4 h; 6 wk-old plants; better photosynthesis parameters) relative to WT under the same conditions. Detached leaves treated with air drying or 10% PEG6000 had greater relative FW if the leaf was from the transgenic plants as compared to the WT. | [231] |
| CaLEA1  [group 5; LEA_3; pfam PF03242]  <GenBank cDNA KC795265> | *Capsicum annuum*  cv. Hanbyul  pepper | In VIGS pepper plants from inoculation of fully expanded cotyledons (despite still detectable transcript), reduced drought (lower survival percentage after 16 d withholding water and 2 d rewatering) and NaCl (lower chlorophyll and higher MDA content in detached leaves in 0.2 M for 7 d) tolerance relative to empty vector control plants. |  | In Arabidopsis ectopically expressing *CaLEA1*, seeds were more sensitive to ABA (1.5 μM) relative to WT seeds as measured by the percentage that completed germination; and by the length of the root for those that did complete germination on a range of ABA concentrations. Three wk-old transgenic plants had enhanced drought tolerance (higher survival percentage after 11 d withholding water and 2 d rewatering) compared to WT plants. Water transpiration was slower from detached leaves and the stomata aperature of detached leaves were smaller in response to ABA for the transgenics relative to WT. Although the effect of NaCl was studied, the figures do not match the statements. | [232] |
| CaLEA6  [group 6]  <GenBank cDNA AF1681681> | *Capsicum annuum*  “hot”pepper |  |  | Superior tolerance to dehydration and NaCl in tobacco variety SR1 ectopically expressing *CaLEA6*, relative to WT. | [233] |
| CaDHN1  [dehydrin; SK_3_-type]  <GenBank cDNA JZ198814; gene JX402924> | *Capsicum annuum* L.  cv. P70  pepper | Reduced cold, NaCl, or mannitol tolerance in pepper, relative to WT. |  |  | [234] |
| CaDHN3  [dehydrin; YSK_2_-type]  <Gene ID CA02g06010 CM334 genome database (http://peppergenome.snu.ac.kr/)> | *Capsicum annuum* L.  cv. P70  pepper | Pepper subjected to VIGS-*CaDHN3* exhibit greater wilting after 24 h at 6 °C with corresponding increased electrical conductivity compared to the vector control plants. Leaf discs from silenced plants subjected to NaCl (0.3 and 0.4 M) or mannitol solutions (0.4 and 0.5 M) had less chlorophyll than those from vector control plants. |  |  | [235] |
| CaDHN4  [dehydrin; Y_3_SK_2_]  <CA02g22060 CM334 genome database (http://peppergenome.snu.ac.kr/)> | *Capsicum annuum* L.  cv. P70  pepper | Pepper subjected to VIGS exhibit decreased seedling growth and reduced cold-and NaCl-tolerance. |  | Arabidopsis ectopically expressing *CaDHN4* displayed superior completion of germination and root growth under NaCl stress and on ABA (various concentrations depending on assay), relative to WT. Under cold and salt stress, the overexpressing plants had lower MDA content and EL compared to WT. | [236] |
| CbCOR15a  [assuming LEA_4; pfam PF02987]  <GenBank cDNA JF718274> | *Capsella bursa-pastoris*  Shepherd’s purse |  |  | Tolerance to chilling (4°C or 0°C) increased in tobacco ectopically expressing *CbCOR15a*, relative to WT. | [237] |
| CbCOR15b  [assuming LEA_4; pfam PF02987]  <GenBank cDNA AY437888) | *Capsella bursa-pastoris*  Shepherd’s purse |  |  | Tolerance to chilling (4°C or -4°C) increased in tobacco ectopically expressing *CbCOR15b*, relative to WT. | [238] |
| CkLEA2-3  [LEA_2]  <GenBank cDNA KU350658> | *Caragana korshinskii* Kom.Korshinsk pea shrub |  |  | In Arabidopsis (Heynh), seed germination occurred faster for overexpressing lines in the presence of 0.2 M NaCl and 0.4 M mannitol compared with WT. Survival percentages after 3 d of rewatering following 10 d of withholding water was greater for the transgenic plants. Seeds from the transgenic plants completed germination faster and to a greater final percentage (13 d) when in the present of ABA (6 μM) as compared to the WT seeds. | [239] |
| CarDHN  [dehydrin; SK_5_-type]  <GenBank cDNA HQ123366> | *Cerastium arcticum* Lange  arctic chickweed |  |  | *S*. *cerevisiae* (BY471) cell with constitutively expressing CarDHN recovered more rapidly (as determined via a spotting assay) than vector control-yeast cells in the presence of exogenous stimuli, including H_2_O_2_, MD, t-BOOH, NaCl, ZnCl_2_, and freezing and thawing, but were more sensitive to CuCl_2_, SDS, and CoCl_2_. Similar results were apparent with the *E*. *coli* (BL21 (DE3)). Under conditions of batch fermentation, the spot assays also determined a greater resistance to ethanol for log phase cells of the transgenics compared to vector controls. [240]  In tobacco (cv. Havana petit) ectopically expressing *CarDHN* tolerance to NaCl increases but not drought tolerance, relative to WT. Seed germination and seedling growth was improved in the presence of NaCl (0.2 M) and mannitol (0.3 M) for the *CarDHN*-OE tobacco plants relative to the untransformed WT. The overexpressing seedlings survived -10 °C treatment with a prior acclimation exposure to 4 °C better than did the WT plants. Drought stress tolerance was no different between the overexpressing and WT plants. Proline quantities were reduced in overexpressing plants compared with WT plants with NaCl (0.2 M) irrigation. [241,242] | [240-242] |
| CpLEA5  [group 5; LEA_3; pfam PF03242]  <GenBank cDNA KT727031> | *Chimonanthus praecox* (L.) Link  Wintersweet or Japanese allspice |  |  | *E*. *coli* (DH5α), ectopically expressing *CpLEA5* have superior cold tolerance, relative to WT. *Pichia pastoris* (GS115) ectopically expressing *CpLEA5* have superior cold, NaCl and NaHCO_3_ tolerance, relative to WT. | [243] |
| hiC6  [48 % identity to LEAs]  <GenBank cDNA U38231> | *Chlorella vulgaris*  (C-27)  green algae |  |  | Increased survival of *S*. *cerevisiae* (INVSc2) expressing *hiC6* after induction after thawing as compared with the vector control yeast. | [244] |
| CbCOR15  [assuming LEA_4; pfam PF02987]  <GenBank cDNA EF208112> | *Chorispora bungeana*  blue mustard |  |  | Tobacco (cv. Xanthi) overexpressing plants exposed to 2 °C for 7 d increased EL but less so than the untransformed control plants so exposed. Chlorophyll and RWCs were greater for the transgenics than the control plants. Further testing with 1 h of 0, -2, -4 and -6 °C and -2 °C for 0.5, 1, 2, 4, 6 and 8 h showed similar reductions in EL compared to the untransformed plants. | [245] |
| CuCOR19  [dehydrin]  <GenBank cDNA AB016809> | *Citrus unshiu*  Marcov.  Satsuma mandarin |  |  | In tobacco (cv. Samsun), ectopic expression of CuCOR19 resulted in superior cold tolerance, potentially due to reduced MDA production at cold temperatures. | [246] |
| pcC6-19  pcC3-06  pcC27-45  [assuming pcC6-19 dehydrin YSK_2_-type; pcC3-06 assuming LEA_4 pfam PF02987; pcC27-45 assuming LEA_2 pfam PF03168]  <cDNAs in Figure 2 [247]; assuming pcC6-19 GenBank gene X74067; assuming pcC27-45 GenBank gene X69883> | *Craterostigma plantagineum*  (Syn. *Torenia plantagineum*)  resurrection plant; blue gem |  |  | Leaf samples of tobacco overexpressing, null segregant and untransformed plants were treated with PEG6000 (30%) to mimic drought stress and then the EL determined; no differences were noted. Similarly, no difference in photosynthetic or respiration rates could be observed between the untransformed or overexpressing plants. | [248] |
| CsASR1  [ASR; pfam PF02496]  <Gene ID Csa005659> | *Cucumis sativus* L.  cv. Chinese long No. 9930  cucumber |  |  | *E*. *coli* (BL21 (DE3)) survived NaCl (0.1, 0.2 M) and sorbitol (0.6 M) when engineered to overexpress CsASR1. Seed from Arabidopsis overexpressing CsASR1 completed germination faster and to a higher final percentage (5 d) in the presence of NaCl (0.1, 0.2 M) than the untransformed-plant seed. NaCl-watering (0.2 M) for 14 d was not as detrimental to the transgenic plants compared to the untransformed plants. | [249] |
| CsLEA11  [dehydrin; Y_3_SK_2_-type]  <GenBank cDNA XM_004150027.2; current.3> | *Cucumis sativus* L.  cv. Chinese long No. 9930  cucumber |  |  | *E. coli* (BL21(DE3)) has enhanced tolerance to supra- and sub-optimal temperature. | [250] |
| CdDHN4-L  CdDHN4-S  [dehydrin; Y_3_SK_2_ type]  <CdDHN4-L GenBank CDS KX243552; CdDHN4-S GenBank CDS KX243553> | *Cynodon dactylon X Cynodon transvaalensis*  cv. Tifway  bermudagrass |  |  | *E. coli* (Rosetta (DE3)) and Arabidopsis overexpressing either DHN were more tolerant of salt (NaCl), cold and heat compared to the control cells/plants. Potentially, the lack of the one segment in the S (shorter alternatively spliced) reduced the tolerance slightly. | [251] |
| DofLEA[17 genes cloned in 7 groups of LEAs]<proteins in Table S1 in [252]> | *Dendrobium officinale*typical epiphytic orchid |  |  | Comparison of *E*. *coli* (BL21DE3)) with the empty vector (pET28) to one containing one of each family identified in the genome were tested in NaCl (high 0.3 – 0.6 M) or heat (50 °C) resulted in 6/7 having a positive correlation to the stress resistance. | [252] |
| EjDHN1[dehydrin]<GenBank cDNA FJ472835> | *Eriobotrya japonica* Lindl.  loquat |  |  | Improved cold stress tolerance in transgenic tobacco ectopically expressing EjDHN; greater survival of exposure to MV relative to WT. | [253] |
| FaASR[ASR] <GenBank cDNA JN006160 in [254];  gene ID 08120 (https: //strawberry.plantandfood.co.nz/index.html in [255]> | *Fragaria × ananassa*  cv. Toyonaka in [254]  cv. Sweet Charlie in [255]  strawberry |  | Immature fruit injected with *Agrobacterium tumefaciens* transformed with ASR-containing vector accumulated more anthocyanin, sucrose and ABA compared with the empty vector control fruits. The firmness of the ASR OE fruits was reduced compared to the empty vector fruits. [255] | In yeast AH109, N-terminal 100 amino acids of FaASR contains an activating domain. [254] | [254,255] |
| PM2[group 3]<GenBank cDNA M80664> | *Glycine max* L. Merr.  cv. Bainong 6#  soybean |  |  | Using spot assays and survival percentages, enhanced NaCl and KCl (0.7 M) tolerance in *E. coli* (BL21 Star (DE3)) ectopically expressing PM2. [256]  The same methods showed greater tolerance to MgCl_2_ and CaCl_2_, and additional to a pulse of cool (24 h 4 °C), freezing (24 h, -20 °C) and warm temperatures (45 min, 50 °C). [257] | [256,257] |
| PM11PM30ZLDE-2[Group 1, 3 and 2, respectively]<PM11 GenBank cDNA AF004805; PM30 GenBank cDNA AF117884; ZLDE-2 GenBank cDNA AY351918> | *Glycine max* L. Merr.  cv. Bainong 6  soybean |  |  | *E*. *coli* (BL21 Star (DE3)) expressing PM11 and PM30 had shorter lag period and improved growth in LB with NaCl (0.8 M), KCl (0.7 M), and upon treatment at 4 °C | [258] |
| GsPM30  [group 3A]  <similar to Glyma13g363300> | *Glycine soja*  wild soybean |  |  | 14 d after completing germination, Arabidopsis overexpressing-GsPM30 resulted in greater tolerance to NaCl (0.2 M, 4d) and water deficit (4 h, rehydrate overnight) as compared to untransformed plants. Prior to flowering, 10 d withholding water and then rewatering for 7 d, overexpression of GsPM30 assisted with survival compared to untransformed plants. See Text and Figures for detailed explanation of a binding partner. | [80] |
| Cot_AD24498  [group 2]  <Undisclosed> | *Gossypium hirsutum*, *Gossypium cuminte* and *Gossypium raimondii*  cotton (Uncertain which was used in transgenic plants.) |  |  | In Arabidopsis, overexpressing-LEA2 in the presence of mannitol (0.1, 0.2 and 0.3 M) for 6 d had longer roots and FW than untransformed controls. Radical scavenging enzymes were upregulated in those drought-treated overexpressing plants relative to untransformed plants. | [259] |
| GhDHN03  GhDHN04  [dehydrin; pfam PF00257]  <GhDHN03 Gene ID Gh_A05G1554; GhDHN04 Gene ID Gh_D05G1729> | *Gossypium hirsutum*, *Gossypium cuminate* and *Gossypium raimondii*  cotton (Uncertain which was used in transgenic plants.) | *Gossypium hirsutum* (cv. TM-1) excised leaf of VIGS for either DHN03 or DHN04 had greater water loss and ion leakage than vector control or untransformed leaves under conditions of drought or salt. The VIGS-plants watered with PEG6000 (17%) or NaCl (0.25 M) had greater H_2_O_2_ but less proline and SOD and CAT enzyme activities than either vector control or untransformed plants. |  |  | [260] |
| HbDHN1  HbDHN2  [dehydrin; SK_2_-type]  <HbDHN1 GenBank CDS KX270749; HbDHN2 GenBank CDS KX270751> | *Hevea brasiliensis*  strain Reran7-33-97  Para rubber tree |  |  | 5 d-old Arabidopsis overexpressing either HbDHN1 or HbDHN2 in the presence of mannitol (0.2, 0.3 M) had longer roots than untransformed plants. 4 wk-old Arabidopsis that went unwatered for 15 d (or upon rewatering) survived to a greater percentage if the transformed with either dehydrin. Ion leakage was less but proline content, APX, SOD, and CAT enzyme activities increased during drought for the transgenic plants compared to the untransformed plants under the same conditions. NaCl (0.1 or 0.15 M) reduced the root lengths of the untransformed plants more so than the DHN-overexpression plants. | [261] |
| HvASR5  [ASR]  <Gene ID MLOC_5645; also TC138659 at the TIGR database> | *Hordeum vulgare* L.  cv. Centauro  barley |  |  | Arabidopsis (Columbia) ectopic expression of *HvASR5* increased both NaCl and drought tolerance. Rice (*Oryza sativa* L. ssp. *japonica* cv. Tainung 67 (TNG67)) ectopic expression of *HvASR5* had increased tolerance to drought and sucrose content in roots; sucrose content decreased in leaves. Transcription changes noted. | [262] |
| DHN3  DHN4  [dehydrin]  <Undisclosed> | *Hordeum vulgare*  barley |  |  | In Arabidopsis (Columbia), either dehydrin conferred enhanced drought tolerance (undefined water withholding and rewatering conditions), as determined by relative water content, as compared to WT plants. | [263] |
| DHN4  [dehydrin]  <GenBank cDNA X15287.1> | *Hordeum vulgare*  ssp. *vulgare*  cv. Himalaya  barley |  |  | The DHN4-overexpressing (with COR78 promoter) potato (cv. Desiree) plants flowered earlier and grew taller. A COR78 promoter to drive DHN4 expression were higher tuber yielding than untransformed potato plants across 5 experiments when drought was imposed at the beginning of tuber initiation. Potato (cv. Desiree) parental plants were better yielding than the transgenic plants under wet and cool field conditions. | [130] |
| HVA1  [group 3]  <Undisclosed> | *Hordeum vulgare*  ssp. *vulgare*  barley |  |  | Creeping bentgrass (*Agrostis stolonifera var. palustris* ‘Crenshaw’) when overexpressing *HVA1* under either one of two different promoters had less water loss under drought conditions as compared with untransformed plants. | [264] |
| HVA1  [group 3]  <Undisclosed> | *Hordeum vulgare*  ssp. *vulgare*  barley |  |  | 4 d-old *Avena sativa* L. (oats; cv. Ogle) seedlings engineered to overexpress HVA1 were more tolerant to NaCl (0.1 M) and mannitol (0.2 M) in culturing conditions compared with untransformed seedlings under the same conditions. [265]  Same transgenic lines (R3) were tested with continuous salt (0.05, 0.1, 0.15, and 0.2 M NaCl) irrigation and resulted in less damage to the transgenic plants compared with untransformed plants. The flag leaf area growth, plant height, panicle length and number of spikelets/panicle root system development, number of kernels/panicle and 1000-kernel weight were parameters less affected in the transgenic plants. [266] | [265,266] |
| HVA1  [group 3]  <Undisclosed> | *Hordeum vulgare*  ssp. *vulgare*  barley |  |  | Using leaf discs from untransformed and HVA-overexpressing *Morus indica* L. (cv. K2; mulberry) plants, the transgenics were more tolerance to salt (0.4 M NaCl) and reduced water (2% PEG6000). The transgenics had greater relative water content, membrane stability, photosynthetic yield, proline content and water use efficiency but less photo-oxidative damage. [267]  Same plants were also seen to be more tolerant to cold stress (10 °C) for 4 d than untransformed plants. A stress-inducible promoter was used to reduce the negative growth effects seen in the previous transgenic plants without any stress. Similar stress resilience was demonstrated with the inducible promoter transgenic plants compared with the previous transgenics. [133] | [133,267] |
| HVA1  [group 3]  <GenBank cDNA CAA31853> | *Hordeum vulgare*  ssp. *vulgare*  cv. Himalaya  barley |  |  | Increased tolerance to water deficit (and better recovery after re-watering) and salt (0.1 M NaCl) conferred by the overexpression of HVA1 in rice (*japonica* cv. Nipponbare) plants (3 wk-old, soil grown) compared to untransformed controls plants. [268]  An inducible, synthetic promoter driving HVA1 in rice plants enhanced root initiation and growth with or without treatment (sorbitol); there was increased water use efficiency. Kitaake and Tainung 67 transgenic rice plants survived NaCl watering better than their respectively parental lines. No yield penalty could be observed. [269] | [268,269] |
| HVA1  [group 3]  <Undisclosed> | *Hordeum vulgare*  barley |  |  | In rice (cv. Nipponbare), overexpressing-HVA1 plants had less membrane leakage, greater relative water content, as well as greater osmotic and water potentials after withholding water gradually for prolonged periods as compared to untransformed controls. | [270] |
| HVA1  [group 3]  <Undisclosed> | *Hordeum vulgare*  barley |  |  | Shoot height, shoot FW and DW under salt (0.2 M NaCl 8 d, flushed 2 d; 3 cycles started with 3 wk-old plants) or water deficit conditions (water withheld for 7d recovery 3 d) of rice (Pusa Basmati 1) were greater for the transgenic plants (either constitutive or inducible expressed HVA1) compared to WT or null segregants. Also, lower electrical conductivity after the salt stress for both constitutive-expressed versus stress-inducible expressed plants compared to null segregants; though this parameter was better for the stress-inducible expressed HVA1-plants. | [271] |
| HVA1  [group 3]  <Undisclosed> | *Hordeum vulgare*  barley |  |  | *S*. *cerevisiae* containing a vector with a GAL1 promoter driving HVA1 demonstrated a shorter lag period upon transfer to NaCl (1.2 M) or KCl (1.2 M) and increased survival of a 24 h-freezing condition upon thawing and plating. | [272] |
| HVA1  [group 3]  <Undisclosed> | *Hordeum vulgare*  barley |  |  | Heterologous expression of HVA1 in spring wheat (*Triticum aestivum* L. cv. Hi-Line) enhanced water use efficiency under moderate water deficit as compared null segregants and untransformed plants. [273]  The same transformants were field tested, including dryland, irrigated, and rainfed conditions, in nine experiments over 6 cropping years; and found to have potential for conferring tolerance under stress conditions. [274] | [273,274] |
| HVA1  [group 3]  <Undisclosed> | *Hordeum vulgare*  barley |  |  | The transgenic wheat (commercial Indian variety CPAN1676) plants produced seeds which completed germination fast and a subsequently had a greater seedling growth rate on mannitol (0.2 M), as compared untransformed controls under the same conditions. | [275] |
| HVA1  [group 3]  <Undisclosed> | *Hordeum vulgare*  barley |  |  | In 3-leaf *Zea mays* (maize; Hi II) plants, salt (NaCl at 0.1 and 0.2 M) tolerance (shoot and root length as well as FW) was increased by overexpression of HVA1 (T3) as compared with untransformed plants of the same stage. | [276] |
| IbLEA14  [LEA_2]  <GenBank cDNA GU369820> | *Ipomoea batatas*  cv. White Star in [277]  sweet potato | Calli with reduced osmotic (PEG6000) and NaCl tolerance. [277] | Calli with enhanced osmotic (PEG6000) and NaCl tolerance. Increased lignin content. [277] | Both ectopically- or oxidative-stress inducibly expression in poplar (*Populus alba* × *P. glandulosa*) results in less damage upon MV treatment, and superior tolerance to drought, NaCl, and 42°C for 24 h. [278] | [277,278] |
| IpASR [ASR]<GenBank cDNA MF680587> | *Ipomoea pes-caprae* L.railroad vine |  |  | ASR as a GST fusion protein in *E. coli* BL21(DE3) cells made the cells more resistant to various abiotic stresses (NaCl, sorbitol, and H_2_O_2_) compared to the cells with the empty GST vector. Using a GAL4BD vector and the *S*. *cerevisiae* strain AH109, the N-terminus of 127 amino acids has an activating domain. In culture media, final germination percentage of seeds (7 d) and root lengths of IpASR overexpressing plants were greater than WT when the media included NaCl (0.1, 0.125, or 0.15 M) or mannitol (0.2, 0.3 or 0.4 M). In ectopically IpASR-expressing Arabidopsis plants, salt (0.15 and 0.2 M NaCl) and withholding water for 9 d and rewatering, permitted greater survival as compared to untransformed plants. | [279] |
| IpDHN  [dehydrin; SK_3_-type; pfam PF00257]  <GenBank cDNA KX426069> | *Ipomoea pes-caprae*railroad vine |  |  | IpDHN as a GST fusion protein in *E*. *coli* BL21(DE3) cells made the cells more resistant to various abiotic stresses (NaCl, sorbitol, and H_2_O_2_) compared to the cells with the empty GST vector. In culture media, final germination percentage of seeds (7 d) and root lengths of IpDHN overexpressing plants were greater than WT when the media included NaCl (0.1, 0.125, or 0.15 M) or mannitol (0.2, 0.3 or 0.4 M). Plants under stress had greater proline and relative water content but less ion leakage and MDA content. | [280] |
| IpLEA[LEA_2; pfam PF03168]<GenBank cDNA MF680612> | *Ipomoea pes-caprae*  railroad vine |  |  | Using a spot assay or growth curves, induction of the His-tagged *Ip*LEA, as opposed to an empty vector control, improved tolerance to NaCl (3-5%), sorbitol (0.8-1.5 M) and H_2_O_2_ (0.7-2 mM) in *E. coli* Rosetta (DE3); as well as a 4 h desiccation at 40 °C. Percentage of completion of germination at 7 d on NaCl (0.175, 0.2, or 0.225 M) or mannitol (0.2, 0.3, or 0.4 M) and root lengths were improved compared with untransformed Arabidopsis. Arabidopsis seedlings in soil were also more tolerant to NaCl, mannitol, H_2_O_2_, methyl-viologen, and drought stress with the overexpression of the LEA as compared with untransformed plants. | [281] |
| JcLEA[group 5]<cDNA in Figure 1 of [282]> | *Jatropha curcas* L.  Barbados nut |  |  | Overexpression of *JcLEA* in Arabidopsis as compared with untransformed plants allowed longer roots under PEG4000 (10 and 15%) treatment and greater survival after drought (14 d) and rewatering (10 d). The relative water and glucose contents were greater than and the EL was less if the plants were transformed compared without transformation. Similar trends were observed for NaCl-treated plants. | [282] |
| LsEm1[group 1; LEA_5; pfam PF00477]<GenBank cDNA KY381602> | *Lactuca sativa*  cv. NY 11  garden lettuce |  |  | *E*. *coli* with a *LsEm1*-containing vector grew faster in the presence of PEG6000 (15%) and NaCl (0.6 M) than those bacteria with an empty vector. Rice (cv. Longjing 24) seeds/seedlings which overexpressed LsEm1 were more tolerant to PEG6000 (10, 20 and 30%) and NaCl (0.1, 0.2 and 0.3 M) in culture as determined by root and shoot lengths and final germination percentage. As plants, there was also greater tolerance to NaCl and drought as determined with RWCs, soluble sugar content, chlorophyll content, and SOD activities. Yield was not detrimentally affected without stress but was greater under drought or salt stress for the transgenic plants compared with untransformed controls. | [283] |
| LLA23 [ASR]  <GenBank cDNA AF077629 in [284]; GenBank gene EU652722 in [285]; undisclosed in [286]> | *Lilium longiflorum* Thunb.  cv. Snow Queen  trumpet lily |  |  | Arabidopsis (Columbia) constitutively overexpressing LLA23 as compared with untransformed control seed completed germination to a greater percentage in the presence of ABA (2 to 5 μM), NaCl (60 mM) and mannitol (0.3 M) as well as in the dark. Greater salt tolerance (stem length and weight; silique numbers) of LLA23-overexpressing plants as compared with untransformed plants was observed; and perhaps drought-tolerance. [284]  Cold (1, 3, and 5 °C) and freezing (-4, -6, and -8 °C) tolerance (shoot length and weight and leaf weight for cold; EL and survival for freezing) for Arabidopsis (Columbia) constitutively expressing *LLA23* was demonstrated compared to untransformed plants. [286]  Arabidopsis (Columbia) constitutively expressing *LLA23* both induced and repressed genes (5-fold) of various functional categories as compared with untransformed plants. [285] | [284-286] |
| LcASR[ASR]<GenBank gene JX291143> | *Litchi chinensis* Sonn.  litchi |  |  | Ectopic expressing in Arabidopsis (Columbia) increased drought tolerance, and activities of SOD, CAT, APX, glutathione reductase with drought. Increased gene expression of *RAB18* and *RAB29* and reduced that of *KAT1*, *KAT2* and *SKOR*. | [287] |
| MdoDHN11 [dehydrin; Y_3_SK_2_-type]  <GenBank cDNA KF578381> | *Malus x domestica* Borkh.  apple |  |  | Transgenic Arabidopsis plants overexpressing *MdoDHN11* were more tolerant to severe (6 wk) water deficit compared to WT plants. | [288] |
| MtCAS31[dehydrin; Y_2_SK_4_-type]<likely GenBank gene XM_013597564.2> | *Medicago truncatula* Gaertn  cv. Jemalong A17  barrel medic |  |  | See Text and Figures for detailed explanations regarding drought tolerance and protein interactions. | [109,110,113] |
| MaASR[ASR]<GenBank cDNA AY628102 in [129]> | *Musa cuminate* L. AAA group Brazilian banana  *Musa cuminate* L. AAA group  cv. Dwarf Cavendish (ITC0002)  banana |  |  | Ectopically expressing MaASR in Arabidopsis enhanced drought tolerance. [289]  Arabidopsis plants overexpressing MaASR were delayed in flowering as determined by more days and rosette leaves until flowering occurred as compared with WT. [129] | [129,289] |
| MpASR[ASR]<cDNA in Figure 1A in [290]; Protein in Figure 2A in [291]> | *Musa paradisiaca* L.  French plantain banana |  |  | Arabidopsis constitutively overexpressing MpASR showed greater drought tolerance (better survival after rewatering) than WT plants under the same conditions. [290]  In either *E. coli* (BL21 (DE3) GST fusion protein) or Arabidopsis plants, overexpressing MpASR increased tolerance to osmotic (0.5 M mannitol) and NaCl (0.5 M) stress as compared to control cells and plants; with lower MDA content but higher soluble sugar content. The transgenic seeds completed germination to a greater percentage on multiple media (ABA, mannitol, NaCl, sucrose and glucose) as compared to WT seeds. [291] | [290,291] |
| MusaDHN-1 [dehydrin; SK_3_-type]  <GenBank CDS JF320824> | *Musa*  spp. L. AAA group  cv. Karibale Monthan  banana |  | MusaDHN-1-transformed banana (cv. Rasthali) were more tolerant to drought (shoot cuttings in PEG8000 [2, 5%] or mannitol [50, 100 mM] media; hardened plants withheld water for 15 d) than control plants. |  | [292] |
| NtLEA7-3  [atypical LEA-like]  <GenBank cDNA EF532409> | *Nicotiana tabacum*  cv. Bright Yellow-2 (BY-2)  tobacco |  | Tobacco BY-2 cells overexpressing NtLEA7-3 are more resistant to chilling stress than the WT cells. | Arabidopsis plants overexpressing NtLEA7-3 are more resistant to freezing (gradual [2 h] to final -10 °C), drought (14 d without watering), and salt (final 0.2 M NaCl) stresses. | [293] |
| *Oe*SCR1  [dehydrin; KS-type]  <GenBank cDNA XM_023012427; protein XP_022868195.1> | *Olea europaea* L.  var. *sylvestris*  olive |  |  | In tobacco (cv. Samsun) transgenic plants enhanced tolerance (higher survival, better shoot and root growth, greater free proline content, and higher APX activity) as compared to WT under cold (3 d 4 °C), NaCl (0.2 M), or drought (germination on 0.4 M mannitol; 20% PEG6000 for proline measurement) stresses. | [294] |
| *Oes*DHN  [dehydrin; SK_2_-type]  <GenBank CDS KR349290> | *Olea europaea* L. ssp. *europaea*  var. *sylvestris*  wild olive |  |  | In Arabidopsis, constitutively overexpressing *OesDHN* resulted in increased tolerance to osmotic stress (25 mM mannitol) as measured by slightly larger leaves as compared to WT leaves under the same conditions. | [295] |
| OpsDHN1  [dehydrin; SK_3_-type; acidic]  <GenBank cDNA HO058650 Gene HM581971> | *Opuntia streptacantha*  prickly pear cactus |  |  | Enhanced freezing tolerance (2 h at -15 °C after acclimation), as assessed by survival after recovery, in Arabidopsis constitutively overexpressing OpsDHN1 as compared with untransformed plants. Without stress and with NaCl (0.15 M) treatment, both sets of plants were similar. See Text and Figures for detailed explanation of a binding partner. | [88] |
| OsASR1  [ASR]  <GenBank cDNA AF039573> | *Oryza sativa* L. ssp. *japonica*  cv. Ilmi  rice |  |  | Tolerance increased to H_2_O_2_, NaCl, heat shock, menadione, copper sulfate, sulfuric acid, lactic acid, salicylic acid, and ethanol in *S*. *cerevisiae* (BY4741) expressing *OsASR1*. | [296] |
| OsASR1  [ASR]  <GenBank cDNA AK062319> | *Oryza sativa* L. ssp. *japonica*  cv. Ilmi  rice |  | In 4 wk-old rice (cv. Ilmi) which were constitutively overexpressing *OsASR1* there was increased tolerance to NaCl (0.2 M) and drought (at 3- and 7-d). Less NaCl and greater proline, soluble sugar, and RWC than WT was determined for the transgenic plants, with overall greater survival. The numbers of closed stomata were greater for the transgenic plants used drought conditions as compared to WT. The transgenic plants were more sensitive to exogenous ABA (2.5, 5 μM). Yields (multiple traits) were greater in the field (3 yr) for the transgenic plants compared with WT. |  | [297] |
| OsASR1  OsASR3  [ASR]  <OsASR1 GenBank cDNA AK119547; OsASR3 GenBank cDNA AK066415; OsASR1 14ETL-06-A16, OsASR3 14ROOT-01-K14 obtained from GreenGene BioTech, Yongin, Korea; > | *Oryza sativa* L. ssp. *japonica*  cv. Nakdong  rice |  | In rice, constitutively overexpressing either *ASR1* or *ASR3* permitted greater survival after 3 d of withholding water and rewatering. The photosynthetic parameter ratio (*Fv*/*Fm*) was greater for the transgenic plants compared with WT under both drought and cold (4 °C). |  | [298] |
| OsASR2  [ASR]  <GenBank partial cDNA EF576160> | *Oryza sativa* L. ssp. *indica*  cv. IRBB13 and Zhonghua 11  long-grained rice | RNAi-OsASR2 plants were less resistant (longer lesions) than WT plants when challenged with bacterial blight (*Xanthomonas oryzae* pv. *oryzae* strain PXO99). | Resistance to *Xanthomonas oryzae* pv. *oryzae* (strain PXO99) and *Rhizoctonia solani* (strain YWK196) were increased by the overexpression of *OsASR2*. Osmotic stress (PEG8000 (15% v/v)) reduced root lengths less in the transgenic plants as compared to WT. The transgenic plants survived drought stress (21 d withholding water, 7 d rewatering) better than WT. |  | [299] |
| OsASR5  [ASR]  <RNAi, overexpression LOC_Os11g06720.1> | *Oryza sativa* L. ssp. *japonica* cv.Nipponbare (RNAi by [300-304]  cv. IRAT109 for overexpression [305]  cv. Dongjing for WT and T-DNA [305]  rice | miRNA regulation (36 families, 44 up-regulated and 68 down-regulated) was noted in the roots of ASR5 RNAi plants as compared with untransformed plants. [300]  *OsASR5*-RNAi plants have greater aluminum and drought sensitivity as well as developmental impacts (15 d more needed to flower, abnormal panicle, and reduced seed and trichome numbers; both ASR1 and ASR5 were downregulated by the construct. [302]  Fourteen of 19 proteins downregulated in these same plants had chloroplast-targeting sequences; a GFP fusion protein localized in rice leaf (dark and light conditions) protoplasts. [301]  The same plants show differential expression of 104 putative target genes, including an ABC transporter, *STAR1*, and a binding motif was identified. [303]  New artificial RNAi constructs specific to ASR5 were introduced to Nipponbare variety and determined that the two genes are compensatory for each other, regulate each other and self-regulate such that these new transgenic plants had no sensitivity to Al. [304]  Dongjing T-DNA insertional mutant plants were more sensitive to drought as compared to the untransformed WT plants under the same conditions (15% PEG6000). [305] | In OsASR5 overexpressing rice, better survival under drought stress via more complete stomatal closure and less stomatal conductance as compared with untransformed plants under the same conditions. Longer roots and shoots noted for the transgenic plants compared to the WT in mannitol (0.25 M) and NaCl (0.15 M), respectively. [305] | *E*. *coli* (BL21) were more tolerant of 0.5 M mannitol if the GST vector also included the OsASR5 as a fusion. Arabidopsis plants ectopically expressing OsASR5 were more tolerant to withholding water (3 wk) as noted by the fresh leaf numbers after rewatering (4 d) as compared to the WT control plants. [305] | [300-305] |
| OsASR6  [ASR]  <LOC_Os01g73250> | *Oryza sativa* L. ssp. *indica*  var. IR64  rice |  |  | Arabidopsis (Columbia) overexpression increased leaf size, inflorescence bolt length, and numbers of siliques and seeds. Root biomass increases (length and density) were greater than aerial changes. Higher conductance, transpiration and photosynthesis rate for the same transgenic plants relative to WT control plants. Greater sensitivity to exogenous auxin for 3 d overexpressing seedlings compared to WT. Larger xylem lumen with thicker annular rings were noted for the transgenic roots compared to the untransformed roots. | [306] |
| OsEm1  [group 1; LEA_5; pfam PF00477]  <Os05g0349800; protein in Figure S1 in [307]> | *Oryza sativa* L.  ssp. *japonica*  cv. undisclosed  rice |  | Overexpression in rice increased the sensitivity to ABA and resistance to osmoticum (0.2 M mannitol), and to some extent salt (0.15 M NaCl). There was increased survival during withholding water and recovery after rewatering for the transgenic plants compared to untransformed plants. Other LEAs were upregulated with drought stress but to a greater extent in the transgenic plants compared with untransformed plants. |  | [307] |
| OsLEA1a  [group 1; LEA_5; pfam PF00477]  <obsolete GenBank NM001048621; when that protein blasted, GenBank protein XP_015629387> | *Oryza sativa* L.  ssp. *japonica*  cv. Zhonghua No.11  rice |  |  | In *E*. *coli* (BL21(DE3)), as a N-terminal His-tagged, thioredoxin fusion protein or as a native protein, OsLEA1a reduced resistance to heat (50 °C), freeze/thaw cycles, NaCl, CuSO_4_, sorbitol and H_2_O_2_. Authors suggestion that “may confer antibacterial activity under abiotic stresses”. | [308] |
| OsLEA3-1  [group 3]  <GenBank cDNA DQ789359; protein in Figure 1 in [128]> | *Oryza sativa* L.  ssp. *indica*  cv. Minghui 6 (according to GenBank)  rice |  |  | Three different promoters used to transform the drought sensitive *japonica* rice Zhonghua 11. Under normal field conditions, reduced fertility and grain yield in T1 generation relative to WT. When a selection made for those 15% that did not show this yield penalty for the constitutive and drought-induced promoter construct-plants, the next two generations showed greater resistance to drought as determined by greater yield (due to primarily spike fertility) compared to both the WT and the *Actin*-promoter construct plants. | [128] |
| OsLEA3-1  [group 3A; LEA 4; PF02987]  <GenBank mentioned cDNA U57641; Os05g46480; but AAC03364 in Figure 1 in [309]> | *Oryza sativa* L.  ssp. *japonica*  cv. Lomello (Both GenBank)  cv. Zhonghua 11 (in [309])  rice |  |  | Ectopic expression in *E. coli* (BL21(DE3)) results in superior tolerance to NaCl (1 M), KCl (1 M), CaCl_2_, (1 M), CuSO_4_ (2 mM), MnCl_2_ (2 mM), sorbitol (1.5 M), heat (50 °C) and UV radiation (254 nm @ 80 μW/cm_2_) relative to the WT. | [309] |
| OsLEA3-2  [group 3]  <GenBank cDNA JQ043381> | *Oryza sativa*  ssp. *japonica*  cv. Zhonghua 11  rice |  |  | When expressing the OsLEA3-2, *S*. *cerevisiae* (strain Y001582) had shorter lag times after transferring to NaCl (1.2 M), KCl (1.2 M), and sorbitol (2 M). After sowing seed on MS media and that with NaCl (0.2 M) or sorbitol (0.2 M), the 9 d-old Arabidopsis with constitutively overexpressing OsLEA3-2 had greater FW and DW than untransformed controls under the same conditions. Transgenic rice seed completed germination slightly faster than WT under NaCl (0.1 M), ABA (10 μM) or PEG6000 (10%). Survival after withholding water and recovery after re-watering was greater for the transgenic plants relative to untransformed plants under the same conditions; potentially grain yield as well. | [310] |
| OsLEA4  [group 4b; LEA_1; pfam PF03760]  <GenBank cDNA AK107973.1> | *Oryza sativa* L.  ssp. *japonica*  cv. Nipponbare (in GenBank)  cv. Zhonghua 11 (in [311,312])  rice |  | Overexpressing plants had greater percentage germination on sorbitol, NaCl, and MnCl_2_ than WT. Root but not shoot lengths of transgenic seedlings under these stressors was also superior to WT. Overexpressing plant survival was also greater than WT during drought, NaCl, or MnCl_2_ treatment. [312] | As a thioredoxin fusion (N-terminal) protein, OsLEA4 in *E*. *coli* (BL21(DE3)) increased survival in NaCl (0.5 M), KCl (0.6 M), MgCl_2_ (0.13 M), and CaCl_2_ (0.13 M), heat (50 °C), to freeze-thaw cycles and UV. [311] | [311,312] |
| OsLEA5  [group 5C; LEA_2; pfam PF03168]  <GenBank cDNA JF776156> | *Oryza sativa* L.  ssp. *japonica*  cv. Zhonghua 11  rice |  |  | In *E*. *coli* (BL21), the thioredoxin fusion OsLEA5 protein increased survival in NaCl (0.5 M), KCl (0.6 M), MgCl_2_ (0.13 M), CaCl_2_ (0.13 M), sorbitol (1.6 M),and to heat (50 °C), freeze-thaw cycles and UV as compared to the empty vector in the same cell line. | [313] |
| OsLEA5  [atypical]  <Gene ID LOC_Os05g50710.1 in [84]> | *Oryza sativa* L.  ssp. *japonica*  cv. Nipponbare  rice | Less sensitive to ABA repression of the completion of seed germination. [84] Poorer survival of drought (20% PEG6000) and salt (0.15 M NaCl) treatment, likely due to less sensitivity to ABA (10 μM). [85] See text and figures for more information. | Better survival of drought (20% PEG6000) and salt (0.15 M NaCl) treatment, likely due to more sensitivity to ABA (10 μM). [85] See text and figures for more information. |  | [84,85] |
| Rab16A  [dehydrin; group 2]  <Undisclosed> | *Oryza* *sativa* L.  ssp. *indica*  cv. Pokkali  salt tolerant rice |  |  | Tobacco (cv. Petit Havana SR1) with expression of the *Rab16A* gene survived, flowered and set seed in NaCl (0.2 M in MS media) whereas untransformed plants could not. Greater osmolyte and antioxidative contents in older transgenic plants treated with NaCl (0.2 M) watering as compared with untransformed plants. | [314] |
| Rab16A  [dehydrin; group 2]  <Undisclosed> | *Oryza* *sativa* L.  ssp. *indica*  cv. Pokkali  salt tolerant rice |  |  | Using the gene from the tolerant variety, the Rab16A protein was introduced to salt-susceptible *indica* rice variety Khitish and was induced with NaCl (0.25 M) treatment. Though still inhibited by the salt, shoot and root length as well as chlorophyll contents were reduced less in the transgenic plants compared to the untransformed plants. Proline content and potassium ion (in the shoots) were also greater. | [315] |
| PgLEA  [group 7]  <GenBank cDNA AY823547> | *Pennisetum glaucum* (L.) R.Br.  pearl millet |  |  | *E. coli* expressing *PgLEA* had superior growth when exposed to NaCl (highest 0.75 M) or supraoptimal temperatures (highest 55 °C). | [316] |
| PpDHNA  [dehydrin]  <GenBank gene AY365466> | *Physcomitrella patens*  Gransden WT isolate  moss | Using targeted gene disruption, null mutant moss were less tolerant of mannitol osmotic stress (0.9 M) or NaCl (0.5 M) stress, as determined by FW, DW and chlorophyll contents upon recovery. |  |  | [317] |
| PpDHNA  PpDHNB  [dehydrin; Y_11_K-type and K_2_-type, respectively]  <DHNA GenBank protein AAR13080.1 or Phypa_221321 from *Physcomitrella patens* ssp patens v1.1; ;  DHNB obsolete GenBank XM 001785041.1; instead Phypa_173331 from *Physcomitrella patens* ssp patens v1.1 > | *Physcomitrella patens*  Gransden WT isolate  moss | Gene-disruption *ppdhna* mutants (from [317]) were more sensitive to osmoticum and salt stresses (repeatability) than the *ppdhnb* mutants, which were only occasionally different than the untransformed moss. [318] |  | As YFP-N-terminal fusion proteins constitutively overexpressed in Arabidopsis, PpDHNA ,but not PpDHNB (for the most part), was able to confer some increased tolerance to osmotic (0.25 M sorbitol; 18% PEG6000) and NaCl (0.1, 0.15 M) stress compared with WT plants. [318] | [317,318] |
| PpDHNA  PpDHNC  [dehydrin]  <PpDHNA Gene ID Phypa_221321; PpDHNC Gene ID Phypa_173172; with Physcomitrella patens ssp patens v1.1 @ https://mycocosm.jgi.doe.gov/pages/search-for-genes.jsf?organism=Phypa1_1> | *Physcomitrella patens*  moss |  |  | In Arabidopsis, both as GFP fusion proteins or native proteins, both moss dehydrins conferred some level of tolerance to both osmotic (0.3 – 0.4 M mannitol) and NaCl (0.15 M) media (7 d) whether constitutively expressed or under their own promoter as compared to untransformed plants. As 3 wk-old plants treated with NaCl (0.2 M) for 4 wk or withholding water for 2 wk, there was also greater survival. Measurements of antioxidant enzyme activities were greater in the transgenic plants compared to WT plants. | [319,320] |
| PpDHNA  PpDHNB  PpDHNC  [dehydrin]  <Undisclosed> | *Physcomitrella patens*  strain Gransden  moss |  |  | Tobacco (variety SR) transformed with PpDHNA and B but not C were more resistant to osmotic (0.2 M sorbitol) stress compared to WT and empty vector control plants. Proline or soluble sugars and MDA or RWCs were positively and negatively correlated, respectively, with the resistance to the stress, most tightly only for PpDHNA transgenic plants. Slightly the same effects for a cold treatment as with withholding water. | [321] |
| PicK  PicM  PicW1  PicW2  [dehydrin]  <Protein in Figure 3 in [322]> | *Picea koraiensis,*  *Picea meyeri, and*  *Picea wilsonii*  Korean spruce,  Meyer’s spruce, and Wilson’s spruce |  |  | Freezing tolerance (-5 or -20 °C) is increased with the presence of any one of the dehydrins from the spruce species (as a chitin fusion protein) in the *E*. *coli* (BL21(DE3)) cells compared with an empty vector containing culture. | [322] |
| PicW  [dehydrin]  <cDNA in Figure 2 and protein in Figure 3 in [323]> | *Picea wilsonii*  Wilson’s spruce |  |  | Tobacco (cv. Xanthium) transformed with the spruce PicW were more tolerant to -5 °C (3 h) treatment; resulting in greater soluble sugar and proline content but less MDA and relative EL as compared to untransformed plants. | [323] |
| PicW  [dehydrin]  <Stated from [323]> | *Picea wilsonii*  Wilson’s spruce |  |  | Though there was no difference at 5 °C, there was enhanced freezing tolerance (-5 °C for 6 h) of creeping bentgrass (Agrostis stolonifera L. ‘Penn A-4’) transformed with PicW as compared with untransformed plants. There was lower MDA and relative EL but higher proline and soluble sugar contents in those stolon node-infected derived plants compared to the WT plants. | [324] |
| PpLEA3.1  PpLEA 3.2  PpLEA 3.3  [group 3]  <all splice variants in GenBank gene GU947646; PpLEA3.1 GenBank partial cDNA GU947647; PpLEA3.2 GenBank CDS GU947648; PpLEA3.3 GenBank CDS GU947649> | *Pogonatherum paniceum*  baby bamboo grass |  |  | In *E*. *coli* (BL21(DE3)) as His-tagged (maybe even thioredoxin) fusions, the PpLEA3 variants decreased the lag time once transferred to NaCl (0.8 M), 4 °C, or 45 °C as compared to empty vector cultures. | [325] |
| PeDHN  [dehydrin; SK_2_-type]  <cDNA in Figure 2 in [326]> | *Populus euphratica*  Euphrates poplar |  |  | *Populus tremula × Populus alba* transgenic plants expressing PeDHN constitutively lost water more slowly (RWC) compared to untransformed plants as the withholding of water continued. | [326] |
| PmLEA8  PmLEA10  PmLEA19  PmLEA20  PmLEA29  [dehydrin; 8 YnKn-type; 10 and 20 YnSKn-type; 19 and 29 SK_3_-type]  <Gene ID Pm026682; Pm026684, Pm020945, Pm021811, and Pm006114, respectively from http://prunusmumegenome.bjfu.edu.cn/> | *Prunus mume* Sieb. et Zucc.  cv. Beijingyudie  Japanese apricot |  |  | *E. coli* BL21 (DE3) transformants of any of 4, but not PmLEA8, dehydrin-GST fusion proteins were more tolerant to osmotic (1 M sorbitol) or freeze-thaw cycling stress relative to the empty vector cultures. Transformants of each of the 4 dehydrin as ectopic expression in tobacco (*N*. *benthamiana*) relative to an empty vector control plants had less EL and MDA content under both cold (4 °C for 24 h) and withholding water for 15 d yet greater RWC. | [327] |
| RcDHN5  [dehydrin; SK_2_-type; acidic]  <GenBank cDNA EU549866> | *Rhododendron catawbiense*  Catawba rhododendron |  |  | Ectopic expression increased freezing tolerance in non-acclimated Arabidopsis plants; decreased the lethal temperature (50 °C) by 0.7 °C and the injury (measured as EL) observed as compared to untransformed control plants. | [328] |
| RcLEA  [group 7]  <protein in Figure 1 in [329]; primers designed for GenBank cDNA BI978651> | *Rosa chinensis* Sohloss Mannieim  China rose |  |  | Expression in *E. coli* results in enhanced NaCl, supra- and sub-optimal temperatures and H_2_O_2_ tolerance. Expression in Arabidopsis results in superior supra-optimal temperature stress (45°C, 24 h) tolerance and greater freezing shock (-20°C, 4 h) recovery. | [329] |
| SbASR-1  [group 7]  <GenBank cDNA EU746399; gene KM462537> | *Salicornia brachiata*  halophyte |  |  | Constitutively expressed SbASR-1 in tobacco (cv. *xanthii*) permitted greater ability of the seeds to complete germination and seedlings to grow upon exposure to NaCl as compared to the untransformed plants. The leaf samples from the transgenic plants had less proline and Na content. [330]  In the first generation, transgenic peanut (*Arachis hypogaea* L.) plants were more tolerant to both salt and the withholding of water. Upon imposing a stress, the transgenic plants had higher chlorophyll and RWCs in their leaves of as compared to those of the untransformed plants; while having lower EL, MDA content, proline, sugars, and starch accumulation. DNA binding was demonstrated with a specific nucleic acid sequence, likely the SbASR-1 acts as a transcription factor. [331] | [330,331] |
| SmLEA  [group 5c; LEA_2; pfam PF03168]  <GenBank cDNA AY725206> | *Salvia miltiorrhiza* Bunge  Chinese sage |  | Growth inhibition under stress (0.1 M NaCl, 0.15 M mannitol and 15% PEG6000) conditions in *S*. *miltiorrhiza* transgenic plants were greater for the empty vector plants as compared to the constitutively overexpressing SmLEA-*S. miltiorrhiza* plants. | In *E*. *coli* (BL21(DE3)) with the SmLEA achieved logarithmic phase 10 h earlier than empty vector when cultured with NaCl (0.8 M). | [332] |
| SmLEA2  [LEA_2; pfam PF03168]  <GenBank partial cDNA HQ676610> | *Salvia miltiorrhiza* Bunge  Chinese sage | Enhanced vegetative water loss over the entire time course, greater MDA accumulation, less SOD activity on 15% PEG6000 for the RNAi-transgenic plants compared with vector control plants. Less consistent but similar trend for the transgenics exposed to NaCl (0.1 M) relative to the empty vector control plants; yet, under salt stress, a lower K^+^ to Na^+^ ratio than the control plant. | Reduced vegetative water loss (at initial time points), less MDA content, and greater SOD activity upon drought stress (15% PEG6000) for ectopic constitutive overexpression as compared to vector control plants. Less consistent results, but a similar trend, with the transgenic plants in NaCl (0.1 M) as compared to control empty vector plants; yet with salt treatment, a higher K^+^ to Na^+^ ratio for the transgenics compared to control plants. | Ectopic expression (pET28) in *E. coli* (BL21) allowed greater tolerance of NaCl (0.4 M solid, 0.8 M liquid) as compared with WT *E*. *coli*. | [333] |
| SiDHN  [dehydrin; YK_2_-type]  <GenBank cDNA KJ145794> | *Saussurea involucrate* Kar. et Kir.  snow lotus |  |  | Tomato (*Solanum lycopersicum* L.) plants when constitutively overexpressing SiDHN were better able to withstand cold (4 °C for 48 h, then 0 °C for 48 h prior to recovery at 25 °C) and drought (24 d withholding water and then rewatered) than WT plants. Even though the plants were shorter and similar FW, the transgenic plants had greater DW under unstressed conditions; mostly attributable to longer roots with greater FW and DW but also thicker stems. Both stresses decreased RWC and increased MDA content and relative EL more so in the WT compared to transgenic plants. Even though the stresses increased proline and soluble sugars, the increases were more pronounced for the transgenic plants compared to the WT. Antioxidant enzyme activities were generally greater for the transgenic plants under stress compared to WT with the same conditions. | [334] |
| SiDHN2  [dehydrin; KS-type]  <GenBank cDNA KF128736> | *Saussurea involucrate* Kar. et Kir.  snow lotus |  |  | Greater freezing (-2 °C for 2 h) and drought (water withheld for 7 or 20 d) in tobacco (*N*. *nudicaulis* (NC98)) tolerance with either constitutive or stress-inducible promoter control of *SiDHN2* as compared to untransformed plants. Higher RWC but lower MDA content for the transgenic plants compared to the WT plants under the same stress. | [335] |
| SiASR1  [ASR; pfam PF02496]  <Gene ID Si011081; protein and CDS, respectively, in Figure S2 and S3 in [336]> | *Setaria italica*  cv. Yugu 1  foxtail millet |  |  | In tobacco (W38) overexpression of the SiASR1 assisted in withstanding drought and oxidative stresses, determined as higher survival rate with RWC, ion leakage, chlorophyll content and antioxidant enzyme activities correlated appropriately compared with untransformed control plants. | [336] |
| SiASR4  [ASR]  <Gene ID Seita.5G463800; protein in Figure 1A in [337]> | *Setaria italica*  cv. Jigu 11  Foxtail millet | RNAi transgenic foxtail millet were more sensitive to growth on MS media containing ABA from a shoot length perspective than the WT. | Overexpressing SiASR4 in foxtail millet permitting greater shoot and root growth relative to WT when the MS media contained NaCl (0.1 M), ABA (5 μM) or PEG6000 (15%). Withholding water (10 to 13 d, then 5 d recovery) from or watering with NaCl-containing water (0.4 M for 20 d) was also less detrimental to the transgenic plants relative to the WT plants. | In yeast, transcriptional activation could not be demonstrated. Although reduced relative to the MS media alone, the number of lateral roots and chlorophyll content was less affected in Arabidopsis overexpression plants in the presence of (0.15 – 0.2 M NaCl). Survival of plants after recovery from withholding water for 15 d was also better for the transgenic plants over the WT control plants. | [337] |
| SiLEA14  [group 5C LEA_2; pfam PF03186]  <GenBank cDNA KJ767551> | *Setaria italica* Beauv.  cv. Jigu11  foxtail millet |  | Seeds in media containing osmotic (10-20% PEG6000), NaCl (0.1-0.2 M) and ABA (10 μM) for 4-9 d had subsequently larger shoots and roots as seedlings if they were SiLEA14-OE transgenic plants compared to untransformed plants. Constitutive overexpression of SiLEA14 enhanced NaCl (0.15, 0.25 M) and drought (7d then recovery 3d) tolerance as plants. Soluble sugar and proline increases were greater for the transgenic plants compared to WT. | Overexpression in *E. coli* resulted in superior NaCl (0.6 M) and KCl (0.6 M) tolerance compared with empty vector control cultures. Decreases in FW were less for transgenic ectopic expressing-SiLEA14 Arabidopsis than untransformed WT in the presence of NaCl (0.125 M) and mannitol (0.25 M). | [338] |
| ShDHN  [dehydrin; SK_3_-type]  <GenBank partial cDNA KF585024> | *Solanum habrochaites*  wild tomato |  |  | Introducing ShDHN into a processing cv. of tomato, LA4024, that is usually chilling sensitive, improved in tolerance to this stress (3d at 4 °C) relative to untransformed plants. Less increase in EL and yet greater increases in proline and soluble sugar contents upon the chilling treatment for the transgenic plants compared to WT. Various responses to the cold stress appears for 4 different antioxidant enzyme activities; overall greater for transgenic plants. | [339] |
| ASR1  [ASR]  <GenBank in [131,340,341] partial gene U86130> | *Solanum lycopersicum*  cv. Ailsa Craig in GenBank  tomato | RNAi -SlASR1 in potato (cv. Desiree) reduced tuber FW compared to untransformed WT potatoes. [131]  Tobacco ‘Xanthi-nc’ with anti-sense constructs of SlASR1 (T4) had reduced height, stem/root/leaf FW, internode length, CO_2_ assimilation and ABA but increased time to flowering and glucose content compared to WT plants. GAs, specifically C20 oxidation and C3 oxidation metabolites, were decreased and increased, respectively, in the AS plants compared with the WT plants. [340]  Tomato (cv. Moneymaker) with an RNAi-construct were variably more sensitive to withholding water (and recovery) compared to WT control plants. [341]  Transient VIGS fruits showed reduced fruit ripening-specific N-glycan processing enzyme, β-D-N-acetylhexosaminidase gene activation. RIPENING INHIBITOR (RIN) is a positive regulator of *ASR1*. [342] | Tomato (cv. Moneymaker) constitutively overexpressing SlASR1 were less sensitive to 22 d of withholding water and subsequent recovery for 17 d as compared to either the WT or the RNAi-transgenic plants; yet did not show any phenotype with NaCl exposure. [341] | Tobacco ectopically expressed ASR1 from tomato lost water at slower rate and were more tolerant to NaCl than untransformed plants. Even with exposure to the salt, transgenic plants accumulated less Na^+^ and proline than the untransformed plants. [343]  Potato (cv. Desiree) overexpressing SlASR1 had fewer tubers and reduced glucose in those tubers compared to the untransformed potato plants [131]  Arabidopsis (no ortholog gene) ectopically overexpressing constitutively SlASR1 had reduced inhibition of seed germination by ABA, glucose (7% w/v), NaCl (0.2 M) or paclobutrazol. DNA binding of a coupling element 1 present in the *abi4* promoter. [344]  Tobacco ‘Xanthi-nc’ constitutively overexpressing SlASR1 were relatively unaffected compared to WT plants. [340]  *E*. *coli* was transformed with constructs of the ASR1 tethered to types of fluorescent reporters and the amount of fluorescence measured to, upon exposure to NaCl, determine the folding of the ASR1. [345] | [131,341-345] |
| LE4  [dehydrin; group 2]  <Elizabeth A. Bray thanked; sequence likely cDNA in Figure 1A in [346]> | *Solanum lycopersicum*  cv. Ailsa Craig  tomato |  |  | *S*. *cerevisiae* (EH13-15) containing a vector with a GAL1 promoter driving LE4 demonstrated a shorter lag period upon transfer to KCl (1.2 M) but not NaCl as compared with an empty vector control culture. Increased survival (nearly two times that of empty vector cultures) of a 24 h-freezing condition upon thawing and plating. | [272] |
| LE25  [LEA_1; pfam PF03760]  <assuming GenBank gene M76552> | *Solanum lycopersicum*  tomato |  |  | Tomato LE25 increases the salt (NaCl; shorter lag when transferred) and freezing-stress tolerance when overexpressed in yeast (*S. cerevisiae* strain EH13-15) under a GAL1 promoter as compared to untransformed cultures. Under normal conditions, as well as with sorbitol (2 M), the LE25-overexpressing cells were slower to grow than the untransformed yeast. | [347] |
| TAS14  [dehydrin]  <GenBank cDNA X51904> | *Solanum lycopersicum*  cv. Rutgers-Marglobe  tomato |  | Tomato (cv. UC82B) constitutively overexpressing TAS14 were more tolerant to both drought and NaCl than compared with null segregants and WT plants. Even as callus upon exposure to NaCl, Na^+^ did not accumulate to the same degree in the transgenic plants compared to the WT. |  | [348] |
| DHN10  [dehydrin; KS-type; group 2]  <GenBank cDNA AF542504 as in [349]> | *Solanum sogarandinum*  wild potato |  |  | In both sense and antisense directions, under an expected cold-inducible promoter, DHN10 was introduced into potato (cv. Irga) plants. Though the transcripts were there, no protein could be detected for the tagged DHN10. Neither the sense or antisense constructs appeared to have any influence on cold-tolerance. [350]  The same approach taken in cucumber was variable between the transgenic events for increasing freezing tolerance. [351] | [350,351] |
| DHN24  [dehydrin; SK_3_-type; group 2]  <GenBank gene AY292655> | *Solanum sogarandinum*  wild potato |  |  | Using the same cold-inducible promoter as for DHN10 above, the DHN24 was introduced into cucumber (cv. Borszczagowski) and were more tolerant to chilling injury (less EL) upon exposure to 2 °C and then 0 °C for 6 h each (having been treated to 4 °C for 24 h prior to the chilling stress). | [352] |
| SbDHN1  [dehydrin; YSK_2_-type]  <GenBank CDS KT865881> | *Sorghum bicolor* cv. B35  sorghum |  |  | Tobacco (variety SR) which constitutively overexpressed *Sbdhn1* were more tolerant to osmotic stress (0.2 M sorbitol; less MDA content, less EL and more soluble sugar) than either untransformed or empty vector control plants. Withholding water for 7 d, osmotic stress and high temperature combined did not kill the SbDHN1 plants but did the other plants. Subjecting extracts of the leaves of overexpressing plants to high heat did not reduce the amount of soluble proteins as the heat did for even additions of BSA or glycerol to extracts for the untransformed plants. | [353] |
| SpDHN1  [dehydrin; SK_3_-type]  <GenBank CDS KJ000690> | *Stipa purpurea*  isolate KIB309 perennial grass on alpine steppe |  |  | Arabidopsis ectopically expressing SpDHN1 had superior completion of germination and root elongation on PEG6000, relative to WT. | [354] |
| SlASR  [ASR]  <GenBank cDNA KC460335> | *Suaeda liaotungensis* K.  succulent annual halophyte |  |  | Transcriptional activation with the expression (GAL4 reporter system) in yeast (strain AH9) could not be demonstrated. Expression in Arabidopsis (Columbia) resulted in greater salinity (0.4 M NaCl), drought (14 d withholding water) and freezing (2 h -20 °C and then recovery) stress tolerance; but no difference in unstressed conditions. | [355] |
| TaLEA  [LEA_3; pfam PF03242]  <GenBank cDNA DQ663481> | *Tamarix androssowii* Litv.  small shrub/tree grows well in arid or saline conditions |  |  | Ectopic expression in tobacco confers greater drought stress (20% PEG6000 for 7 d) tolerance (less increase in electrical conductivity and MDA content after stress) as compared to untransformed plants. [356]  Ectopic expression in Xiaohei poplar (*Populus simonii* × *P. nigra*) confers greater salinity (0.2 M NaCl for 6 d) and drought (withholding water for 7 d) stress tolerance as determined by less increase in MDA content and EL after stress as compared to untransformed WT control plants. [357] | [356,357] |
| TaLEA3  [Group 5B; LEA_3]  <Same as TaLEA according to Introduction in [358]> | *Tamarix androssowii* Litv. small shrub/tree grows well in arid or saline conditions |  |  | Amur cork tree (*Phellodendron amurense* Rupr. (Rutaceae)) transgenic plants ectopically expressing TaLEA3 increased drought tolerance by faster stomatal closure, likely due to increased calcium uptake and potassium efflux upon PEG6000 exposure. | [358] |
| TaLEA (+ ThbZIP)  [[356] for TaLEA]  <[356] for TaLEA> | *Tamarix androssowii* Litv.  salt cedar  (*Tamarix hispida* Willd. for bZIP)  (Kashgar tamarisk for bZIP) |  |  | Co-expression (constitutively) with *ThbZIP* in birch (*Betula platyphylla*) confers greater salinity stress (4 g L^-1^ for 8 d) tolerance for 2 of 3 transgenic trees. Those two trees, as compared to the non-transgenic or non-resilient tree, after stress, had lower MDA and Na^+^ contents but greater chlorophyll fluorescence, photosynthetic parameters, and some antioxidant enzyme activities. | [359] |
| TtASR1  [ASR]  <GenBank CDS MH454101> | *Tetragonia tetragonoides* (Pall.) Kuntze (Aizoaceae) New Zealand spinach |  |  | No transcriptional activity in yeast (GALT7 strain AH109) was observed. Yeast (pYES strains W303 WT; *yap1Δ* and *skn7Δ* H_2_O_2_-sensitive strains), *E*. *coli* (BL21(DE3); GST fusion) and Arabidopsis (Col-0) were more tolerant to NaCl (pYES 1 M; *E*. *coli* 3%; Arabidopsis seed in MS media, 0.3 M), oxidative (H_2_O_2_ pYES, 0.6 mM; *E*. *coli*, 0.5 mM), osmotic (*E*. *coli* 0.7 M sorbitol; Arabidopsis seed 0.3 M mannitol), and drought (Arabidopsis plants 7 d withholding water and 7 d rewatering recovery) stress conditions. Plants were phenotypical normal under unstressed conditions even with overexpression. | [360] |
| TsLEA1  [group 4B; LEA_1; pfam PF03760]  <GenBank cDNA EU365627> | *Thellungiella salsuginea* (Currently, *Eutrema halophilum*)Shandong ecotypesalt cress |  |  | *S*. *cerevisiae* (strain G19) and Arabidopsis (Columbia) seeds ectopically expressing *TsLEA1* had superior tolerance to NaCl (0.4 M for GAL4 reporter system in yeast; 0.1 M in MS media for seed) than WT organisms. | [361] |
| TaASR1  [ASR]  <GenBank CDS HQ287799> | *Triticum aestivum* L.  cv. Chinese spring  wheat |  |  | Ectopic expression in tobacco increased drought tolerance compared to WT plants. Greater RWC, less EL, and less MDA content for the transgenic plants after drought stress. Greater SOD and CAT activities with corresponding less H_2_O_2_ accumulation found in transgenic plants compared with WT. | [362] |
| TaDHN1  TaDHN2  TaDHN3  [dehydrin; YSK_2_-type]  <TaDHN1 GenBank cDNAs KT633578; TaDHN2 GenBank cDNA KT633579; TaDHN3 GenBank cDNA KT633580> | *Triticum aestivum* L.  cv. SR3  (amphiploid; somatic hybrid of wheat  cv. JN177 x *Thinopyrum ponticum*) |  |  | Expressed from their own promoters in Arabidopsis, only TaDHN1 or TaDHN3 had no yield penalty but had greater tolerance to salt (0.1 M NaCl), osmotic (0.15 M mannitol) and drought stresses. | [363] |
| Em  [group 1]  <GenBank gene X52103 referenced for cDNA clone> | *Triticum aestivum* L.cv. Chinese Springwheat |  |  | *S*. *cerevisiae* (strain INVSc1) ectopically expressing the wheat Em were more resistant to sorbitol-, NaCl- and KCl-stress than WT. | [364] |
| PMA80  PMA1959  [group 2 and 1, respectively]  <Dr Kay Walker-Simmons thanked for plasmids PMA80 and PMA1959> | *Triticum aestivum* L.wheat |  |  | Ectopic overexpression in rice (cv. TNG67) resulted in greater NaCl (0.2 M for 9 d) or drought (8 d withholding and 8 d recovery) tolerance compared with WT plants under the same conditions, as determined by survival, plant height and ion leakage. | [365] |
| WCI16  [LEA-like; “hydrophilic protein reminiscent of LEAs”]  <GenBank cDNA AB830333> | *Triticum aestivum* L.  cv. Chihoku  winter wheat |  |  | When constitutively overexpressed in Arabidopsis (Columbia), WCI16 increased survival at -10 °C and lower EL even without prior acclimation and with no difference to WT plants under optimal conditions. | [366] |
| WCOR15  [undiscussed]  <GenBank gene AB095006> | *Triticum aestivum* L.cv. Mironovskaya 808winter wheat |  |  | As a GFP fusion constitutively or under its own promoter in tobacco (cv. Petit Havana), WCOR15 increased the transgenic plant survival freezing (-10 °C for 2–6 h in the dark) after prior acclimation (4 °C for 3 d). | [367] |
| WCS19  [group 3-related; LEA3-L2]  <GenBank cDNA L13437 referenced in [368]> | *Triticum aestivum* L.cv. Norstarwheat |  |  | Ectopic and constitutive overexpression in Arabidopsis provided modest freezing temperature tolerance compared with WT if acclimated previously. | [369] |
| WZY2  [dehydrin; YSK_2_-type; group 2]  <GenBank cDNA EU395844> | *Triticum aestivum* L.  cv. Zhengyin1  drought sensitive wheat | RNAi plants (of the same cultivar from which the gene had been isolated) were more sensitive to drought stress (5 d first symptoms in RNAi plants, rewatered after 20 d); decreases in chlorophyll were greater for the RNAi plants relative to the WT under the same conditions; but increases in proline, SOD activity and POD activity were less pronounced. Greater accumulation of MDA also occurred in the RNAi transgenic plants relative to WT plants. [370] |  | Using a deletion series of constructs (pET 28) removing each of the motifs in turn, the K segment in *E*. *coli* (BL21(DE3)) was of the most importance to cold (0 °C) and heat (50 °C) tolerance. [371]  Ectopic expression In Arabidopsis (Heynh.) reduced the stomatal aperature under drought conditions (10 d withholding water) or with ABA treatment as compared to empty vector or WT control plants. Proline content, SOD activity and POD activity were higher under drought stress compared with either control plant types; but MDA content was lower. [370] | [370,371] |
| WZY3-1  [group 3 LEA]  <GenBank cDNA KX090360; GenBank protein APJ36638.1> | *Triticum aestivum* L. bread wheat |  |  | In *E*. *coli* (BL21), ectopic expression increased mannitol and NaCl tolerance. In Arabidopsis, ectopic expression increased drought tolerance. | [372] |
| TtASR1  [ASR]  <GenBank partial gene KX660742; CDS KX660744> | *Triticum turgidum* L.  ssp. *durum*  cv. Mahmoudi  salt-tolerant durum wheat |  |  | Ectopically expressed as a GST fusion in *E. coli* (BL21(DE3)), TlASR1 increased both cold (4 °C for 24 h) and heat (50 °C for 45 min) stress tolerance; even under optimal growth conditions, growth was faster for the TtASR1 cultures compared to the empty vector control cultures. *S*. *cerevisiae* (WT strain, KT1112; salt-sensitive strain, KT1623) had better survival upon exposure to NaCl (1 M) or mannitol (0.5 M) when TlASR1 was part of the transforming pYES2 vector included. | [373] |
| DHN-5  [dehydrin YSK_2_-type; pfam PF00257 in [374]]  <GenBank cDNA AY619566> | *Triticum turgidum*  ssp. *durum* durum wheat |  |  | Arabidopsis (Columbia) expressing DHN-5 (as a GFP fusion) were more tolerant than WT to mannitol, NaCl, and LiCl stress. [375]  Transcriptomic alterations include upregulation of many *LEAP*s, downregulation of jasmonate sensitivity. [375,376]  In *E*. *coli* increased tolerance to various abiotic stresses. [377]  Greater salt (0.1 M NaCl) tolerance of Arabidopsis plants ectopically expressing the DHN-5 as a GFP fusion, likely a result of greater antioxidant enzymatic activities and regulation of proline content; though subsequently, also increased protease activities. [378,379] | [375-379] |
| XsLEA1-8  [group 4; LEA_1; pfam PF03760]  <GeneID Xvis02_20008 as in [5]; no sequences per se> | *Xerophyta schlechteri*  monocotyledonous resurrection plant |  |  | Trials of the empty pDEST17 vector versus 6 different XsLEA clones (all as N-terminal His-tagged fusions) for tolerance to NaCl (0.25 M), mannitol (0.35 M) or heat (50 °C) demonstrated the XsLEA1-8 clone as the most resilient to NaCl though it also showed enhanced growth under optimal conditions. Ectopic constitutive overexpression of XsLEA1-8 in Arabidopsis was variable between transgenic plants as compared to untransformed controls for tolerance (root length) to growth when sown as seed on NaCl (0.1 M) or mannitol (0.2 M) in MS media. | [380] |
| ZmASR1  [ASR]  <GenBank assumed cDNA AX297905> | *Zea mays*  ssp. *mays*  cv. MBS847  maize |  | Field evaluation of the transgenic maize inbred A188 under normal and water limited conditions compared to WT plants were greater kernel yields; correlated in part with decreases in branched chain amino acid metabolite, protein, and transcript amounts. |  | [381] |
| ZmASR1 to 9  [ASR; pfam PF02496]  <ASR2 Gene ID GRMZM5G854138_T02; ASR3 Gene ID GRMZM2G044132_T02 in Table S1 in [382]> | *Zea mays*  ssp. *mays*  maize |  |  | Using yeast, the ZmASR3 and ZmASR4 both appear to form homodimers. Arabidopsis seeds from plants constitutively expressing each of the 9 maize ASR1 members were tested on media with mannitol and ABA. Only ASR2 and 3 transgenic plants exhibited longer roots. ASR3 was investigated in greater detail. With mannitol treatment, decreases of root length were less evident for the ZmASR3 transgenic plants relative to WT. Withholding water and then rewatering was also less impactful to the transgenic plants compared to WT, likely with greater stomal closure. Antioxidant mechanisms were much greater for the overexpressing transgenic compared to WT. | [382] |
| ZmASR1 to 10  [ASR]  <ASR1 Gene ID GRMZM2G136910_T01; ASR2 Gene ID GRMZM5G854138_T02; ASR4 Gene ID GRMZM2G168552_T01; ASR5 Gene ID GRMZM2G052100_T01> | *Zea mays*  ssp. *mays*  maize |  |  | ZmASR 1, 2, 4 or 5 in Cd-sensitive yeast *Dycf1* in pYES vector were tolerant to 0.1 mM CdCl_2_, growing even better than WT yeast. Even transient expression of those same 4 ASRs in *N*. *benthamiana* were more Cd tolerant as determined by smaller lesion with subsequent infiltration of Cd solution as compared with an empty vector treatment. | [383] |
| ZmDHN13  [dehydrin; KS-type; group 2]  <GenBank NM_001156643.2; Gene ID GRMZM2G448511> | *Zea mays*  ssp. *mays*  maize |  |  | Ectopic expression in *Pichia* yeast (GS115) copper tolerance (150 mM CuCl_2_) was increased with the ZmDHN13-containing vector as compared with the empty vector; no difference was seen under optimal conditions. Overexpression of the ZmDHN13 in *N*. *benthamiana* resulted in less EL, MDA and superoxide radical content as compared to WT plants, 1 and 3 d after exposure to Cu (70 μM CuCl_2_). | [384] |
| ZmLEA3  [group 3A]  <GenBank gene NM_001153473; Gene ID GRMZM2G096475> | *Zea mays*  ssp. *mays*  cv. Zhengdan958  maize |  |  | Tobacco (cv. NC89) seed constitutively expressing ZmLEA3 completed germination to a greater percentage on MS media containing mannitol (0.2, 0.25 M) as compared to WT seed after 10 d. Less EL, MDA and superoxide radical content were apparent in the transgenic plants. H_2_O_2_ in the media also resulted in less EL for the transgenic plants compared with WT. Yeast (GS115) transformants showed similar resistance to mannitol and H_2_O_2_. [385]  Additional screening of these same tobacco and yeast transformants with low temperature (12 °C) were shown to have resistance. Also, *E*. *coli* (pET30 in BL21(DE3)) showed this resistance. [386] | [385,386] |
| ZmLEA5C  [group 5C; LEA_2; pfam PF03168]  <GenBank gene obsolete NM_001155750.1; truncated would match Figure 1a in [387]; likely Gene ID GRMZM2G017991_T01> | *Zea mays*  ssp. *mays*  cv. Zhengdan958  maize |  |  | As with ZmLEA3, the ZmLEA5C-overexpressing *N*. *benthamiana* and yeast (GS115) were tolerant to osmotic (20% PEG6000 for 5 d) and low temperature (4 °C for 24 h) stresses. | [387] |
| ZmLEA14tv  [group 5C; LEA_2; pfam PF03168]  <GenBank cDNA two nt differences NM_001159174 (Gene ID GRMZM2G050607) but no amino acid changes according to [388]> | *Zea mays*  cv. Tevang 1  maize |  | Constitutive overexpressing ZmLEA15tv maize (K7) seed completed germination to a greater extent than WT seeds in the presence of PEG ?000 (15 and 20%). Five leaf transgenic as compared to WT plants survived to a greater extent withholding water (14 d) and rewatering (3 d). | Tobacco (cv. K326) constitutively expressing ZmLEA14tv recovered more fully than the WT plants after rewatering (3 d) following 15 d of withholding water. | [388] |
| Rab17  [dehydrin; YSK_2_-type; group 2]  <presumably Gene ID GRMZM2G079440> | *Zea mays*  ssp. *mays*  maize |  |  | When constitutively expressed in Arabidopsis (ecotype RLD), survival was greater than WT plants upon exposure to salt (0.2 M NaCl or KCl). The transgenic plants also recovery from mannitol treatment (0.2 M) faster than the WT. | [389] |
| Rab28  [group 5]  <presumably Gene ID GRMZM2G472236> | *Zea mays*  ssp. *mays*  maize |  | Plants constitutively overexpressing *Rab28* in maize (Hi-II genotype [A188 × B73]) were no different than WT under normal conditions, though there were fewer seeds from the transgenic plants. Osmotic stress (10-d plants transferred to 15% PEG8000 media for 5 d) was less severe for the transgenic plants compared to WT, regarding plant size, leaf rolling extent, decreases in relative water, chlorophyll and MDA contents. |  | [132] |
| ZnASR1  [ASR]  <GenBank cDNA KC816463> | *Ziziphus nummularia* (Burm.f.) Wight & Arn  wild jujube |  |  | *E*. *coli* (BL21(DE3)) expressing the ASR (using a pET28a vector) multiplied longer in 10% PEG6000-containing media than the vector control cultures. | [390] |

1: https://www.uniprot.org/taxonomy/ used for common name if provided; otherwise a Google search.; Undisclosed refers not finding a reference number for a sequence or figure with the sequence to report.

Abbreviations in this table: ABA: abscisic acid; APX: ascorbate peroxidase; CAT: catalase; DW: dry weight; *E. coli*: *Escherichia coli*; EL: electrolyte leakage; FW: fresh weight; GAs: gibberellic acids; GFP: green fluorescent protein; MDA: malondialdehyde; MV: methyl viologen; PEG: polyethylene glycol; PEG?000: when the MW of the PEG was undisclosed; POD: peroxidase; ROS: reactive oxygen species; RWC: relative water content; SOD: superoxide dismutase; VIGS: Virus induced gene silencing; and WT: wildtype.
